# The AHEPA EEG benchmark: setting the standard for machine learning in dementia diagnosis, a scoping review

**DOI:** 10.1007/s11571-026-10464-w

**Published:** 2026-05-18

**Authors:** Andreas Miltiadous, Aimilia Ntetska, Vasileios Aspiotis, Efthalia Moustakli, Markos G. Tsipouras, Alexandros T. Tzallas, Nikolaos Giannakeas, Euripidis Glavas, Pantelis Angelidis, Katerina D. Tzimourta

**Affiliations:** 1 Department of Informatics and Telecommunications, University of Ioannina, Kostakioi, Arta, 47100 Greece; 2 Department of Electrical and Computer Engineering, University of Western Macedonia, Kozani, 50100 Greece; 3 Department of Nursing, School of Health Sciences, University of Ioannina, Ioannina, 45110 Greece

**Keywords:** Electroencephalography, Machine Learning, Alzheimer's Disease, Public Dataset, Benchmark, AHEPA

## Abstract

Accurate and reproducible electroencephalography (EEG)-based classification of dementia remains a key challenge in computational neurodiagnostics. The open-access AHEPA dataset has become the most commonly used benchmark for Alzheimer’s disease (AD) and Frontotemporal dementia (FTD) classification, yet reported results vary widely due to methodological inconsistencies. This study presents the first systematic and quantitative benchmark review of all published machine learning approaches applied to the AHEPA dataset. Forty-six studies were reviewed and stratified into three validity tiers, with Validity 1 representing the highest methodological rigor and Validity 3 the lowest.According to their evaluation rigor: (1) subject-level validation (e.g., Leave-One-Subject-Out cross-validation, LOSO-CV), (2) subject-level train/test splits, and (3) epoch-level k-fold cross-validation. Performance metrics were normalized across classification problems. The analysis revealed that methodological rigor is inversely correlated with reported accuracy: for AD versus Cognitively Normal controls, mean accuracy decreased from 90.81% overall to 82.11% in Validity-1 studies; for FTD versus controls, accuracy dropped from 86.53% to 75.18%. Linear regression analyses demonstrated that weaker validation protocols were associated with systematic increases of 7–10% points in reported accuracy, explaining more than half of the observed performance variance. Deep and hybrid models reported the highest nominal accuracies, but under proper validation, traditional algorithms performed comparably, indicating that data leakage often drives apparent improvements. The review also highlights the lack of cross-configuration generalization and the urgent need for adaptive, montage-independent methodologies. Overall, this benchmark establishes the first reproducible reference framework for EEG-based dementia classification on the AHEPA dataset, providing quantitative baselines and validity criteria against which all future studies should be evaluated.

## Introduction

Alzheimer’s disease (AD) is a progressive neurodegenerative disorder and the most common cause of dementia worldwide. It is characterized by the abnormal accumulation of amyloid-β plaques and neurofibrillary tangles of hyperphosphorylated tau protein, resulting in synaptic dysfunction, neuronal loss, and widespread cortical atrophy (DeTure and Dickson 2019). Clinically, AD manifests with memory impairment, executive dysfunction, language and visuospatial difficulties, and, in advanced stages, profound cognitive decline accompanied by behavioural and psychiatric symptoms (Miltiadous et al. 2023a, b, c). The disease course is invariably fatal, with patients experiencing progressive deterioration in functional independence until death (İş et al. 2025).

Epidemiological data indicate that 55–57 million people currently live with dementia, of which AD accounts for approximately 60–80% of cases (2024 Alzheimer’s Disease Facts and Figures, 2024). In the United States alone, an estimated 6.9 million individuals aged 65 years and older are living with Alzheimer’s disease in 2024, and global projections suggest that the total number of people affected by dementia may reach nearly 139 million by 2050. Age is the strongest risk factor, but genetic predisposition (e.g., APOE ε4 allele), cardiovascular comorbidities, and lifestyle factors also contribute significantly to disease onset and progression (Licher et al. 2019). Importantly, there is currently no cure for AD, and available treatments only provide modest symptomatic relief without altering the underlying neurodegenerative process. Beyond its devastating personal toll, AD imposes an enormous socioeconomic burden: the annual global cost of dementia care is estimated to exceed 1.3 trillion USD, a figure expected to rise steeply in coming decades (Wimo et al. 2023).

The current diagnosis of AD relies primarily on a combination of clinical assessment, neuropsychological testing, neuroimaging, and biomarker analysis. In routine practice, physicians evaluate patients based on medical history, cognitive examinations such as the Mini-Mental State Examination (MMSE) and the Montreal Cognitive Assessment (MoCA), and functional assessments of daily living (Wang et al. 2022). Structural magnetic resonance imaging (MRI) and positron emission tomography (PET) can reveal characteristic patterns of cortical atrophy and hypometabolism, while cerebrospinal fluid (CSF) biomarkers such as amyloid-β42, total tau, and phosphorylated tau are increasingly used to support the diagnosis (Márquez and Yassa 2019). However, these methods are costly, invasive, or not widely available, limiting their feasibility for large-scale or early screening. As a result, diagnosis often occurs at relatively advanced stages of the disease, when significant neuronal damage has already taken place and therapeutic interventions are less effective (Bradford et al. 2009). This delay in detection represents a major obstacle to the development of preventive or disease-modifying treatments, as early intervention is widely regarded as essential to preserving cognitive function (Dubois et al. 2023). Furthermore, access to advanced biomarker assays and neuroimaging varies substantially across healthcare systems, creating disparities in diagnostic accuracy and timeliness. Collectively, these limitations underscore the urgent need for affordable, accessible, and non-invasive tools that can enable earlier and more reliable identification of AD.

Given that conventional diagnostic markers are frequently expensive, invasive, or impractical for population-level screening, electroencephalography (EEG) emerges as a feasible and scalable alternative. EEG is a non-invasive technique that records electrical brain activity through scalp electrodes with high temporal resolution and relatively low operational cost, which makes it suitable for repeated measurements and large-scale studies. Over the past decades, quantitative EEG (qEEG) research has highlighted several reproducible alterations associated with AD. Spectral analyses consistently show an increase in power at slower frequencies (delta and theta bands) accompanied by a reduction in alpha and beta activity (Chetty et al. 2024). These shifts reflect a general slowing of brain rhythms and are thought to arise from disrupted synaptic communication and cortical network degradation (Smailovic and Jelic 2019). More refined indices, such as band power ratios (e.g., beta/theta or alpha/theta), have been proposed as reliable biomarkers for distinguishing patients with AD or mild cognitive impairment (MCI) from Cognitively Normal (CN) individuals (Zandbagleh et al. 2024). Beyond simple power spectra, EEG connectivity measures have gained particular attention. Analyses of coherence, phase lag index, and graph-theoretical metrics consistently indicate impaired functional connectivity in AD, especially within the alpha band (Khaleghi et al. 2024). Such findings align with the broader concept of AD as a disorder of large-scale network disintegration rather than isolated regional dysfunction. In addition, emerging approaches explore complexity metrics such as entropy, fractal dimension, and microstate analysis, which can capture subtler aspects of neural dynamics (Miltiadous et al. 2023a, b, c). Taken together, EEG-based biomarkers provide a window into large-scale functional network alterations in AD. However, to realize its potential, the field requires standardized feature extraction pipelines, robust validation on large open-access datasets, and benchmarking across Machine Learning (ML) methodologies to ensure reproducibility and clinical utility.

The integration of ML into AD research provides a powerful framework for accelerating screening and improving diagnostic accuracy. Manual inspection of EEG signals cannot reliably capture the complex and distributed patterns that reflect early neuropathological changes. In contrast, ML pipelines can automatically process EEG data and perform classification with high efficiency. A typical workflow begins with preprocessing steps, such as band-pass filtering, artifact removal (e.g., Independent Component Analysis (ICA) or Artifact Subspace Reconstruction (ASR), and segmentation into epochs (Miltiadous et al. 2023a, b, c). From these epochs, features are extracted, typically including spectral, connectivity, and nonlinear measures as described above. These features are then used as inputs to supervised learning algorithms. EEG classification studies employ both classical machine learning algorithms (e.g., SVM, RF, k-NN, LR) and deep learning architectures, including CNNs, recurrent models (RNNs/LSTMs), and graph neural networks (GNNs), often combined in hybrid approaches to leverage complementary strengths.

**Fig. 1:**
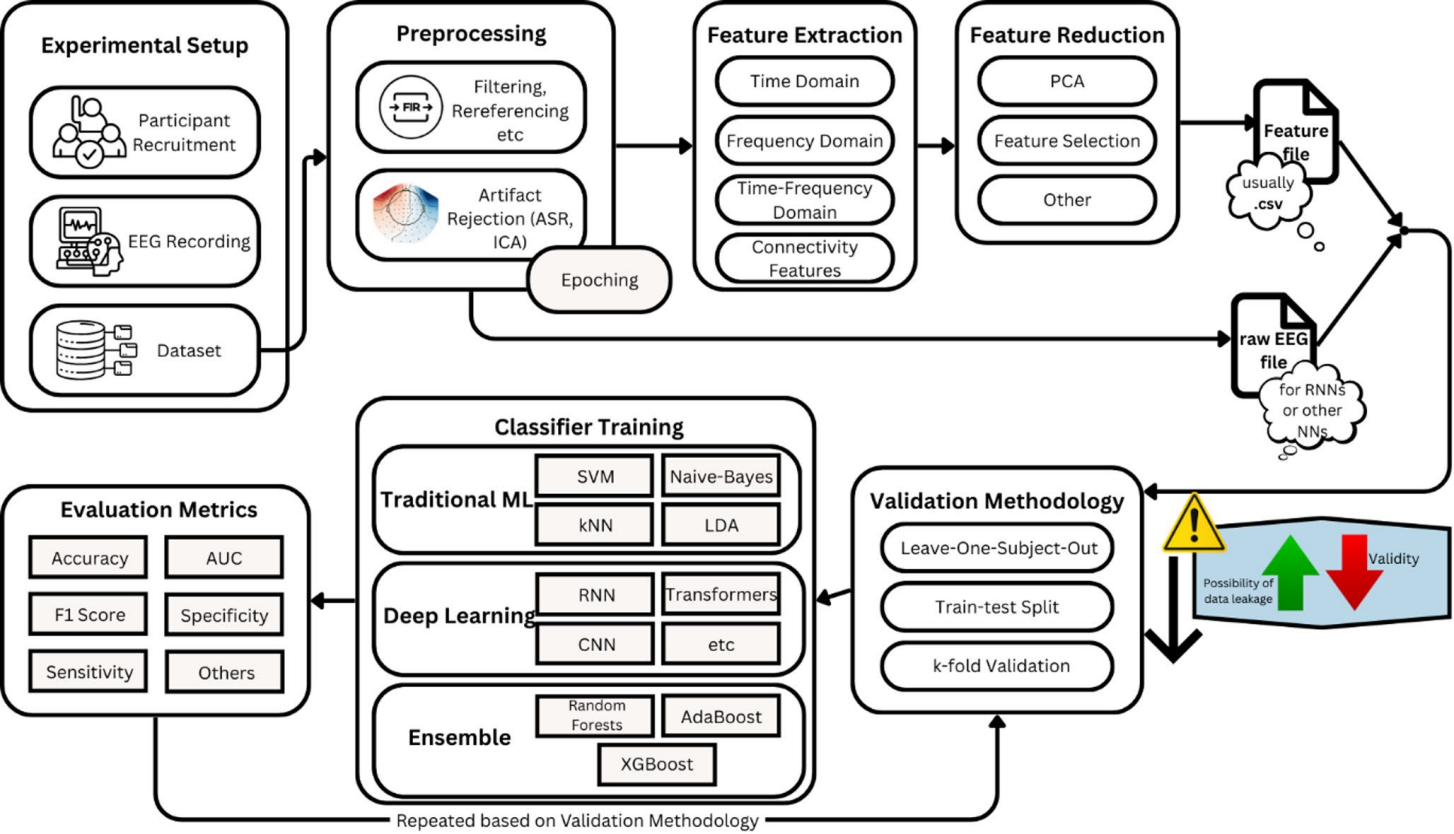
General methodological framework for EEG-based machine learning classification in dementia research

Crucially, the same methodological framework is applicable not only to AD but also to the classification of other dementia syndromes. For example, Frontotemporal Dementia (FTD) exhibits distinct alterations in EEG connectivity and spectral profiles, enabling ML-based differential diagnosis between AD and FTD or between dementia and CN controls. This flexibility underscores the broader potential of ML-driven EEG analysis as a scalable diagnostic technology for neurodegenerative diseases. As depicted in Fig. 1, most EEG-based ML studies follow some or all of these methodological steps: raw EEG recordings are preprocessed and segmented into epochs, features are extracted or directly learned from the data, and classification is performed using either traditional machine-learning algorithms or deep neural networks. The resulting models are then evaluated through common metrics—such as accuracy, F1-score, sensitivity, and specificity—under various validation strategies, including train–test splits, k-fold, or leave-one-subject-out cross-validation (LOSO-CV).

One of the most critical obstacles to advancing ML–based screening for AD is the limited accessibility of suitable datasets. In most cases, research groups rely on data acquired locally from hospitals or memory clinics. Access to such clinical EEG recordings is heavily restricted by privacy regulations, ethical approvals, and logistical challenges related to data sharing. As a result, datasets remain fragmented, with small sample sizes and heterogeneous acquisition protocols. This fragmentation makes it extremely difficult to compare the performance of different methodologies, as the results obtained from one dataset cannot be directly benchmarked against those from another. Consequently, a large portion of the reported performance differences in the literature may reflect dataset-specific biases rather than genuine methodological advances.

To overcome these limitations, the field urgently requires the development and dissemination of open-access databases. Publicly available datasets not only facilitate transparency and reproducibility, but also enable fair benchmarking of algorithms under standardized conditions. Unfortunately, despite the rapid growth of research in this area, only a handful of open EEG datasets for dementia currently exist. Repositories such as OpenNeuro have established themselves as central platforms for hosting and distributing neuroimaging data, providing standardized formats (e.g., BIDS) and encouraging data reuse. Other repositories, such as PhysioNet and Zenodo, have also begun hosting EEG datasets, though coverage of AD remains limited.

**Fig. 2:**
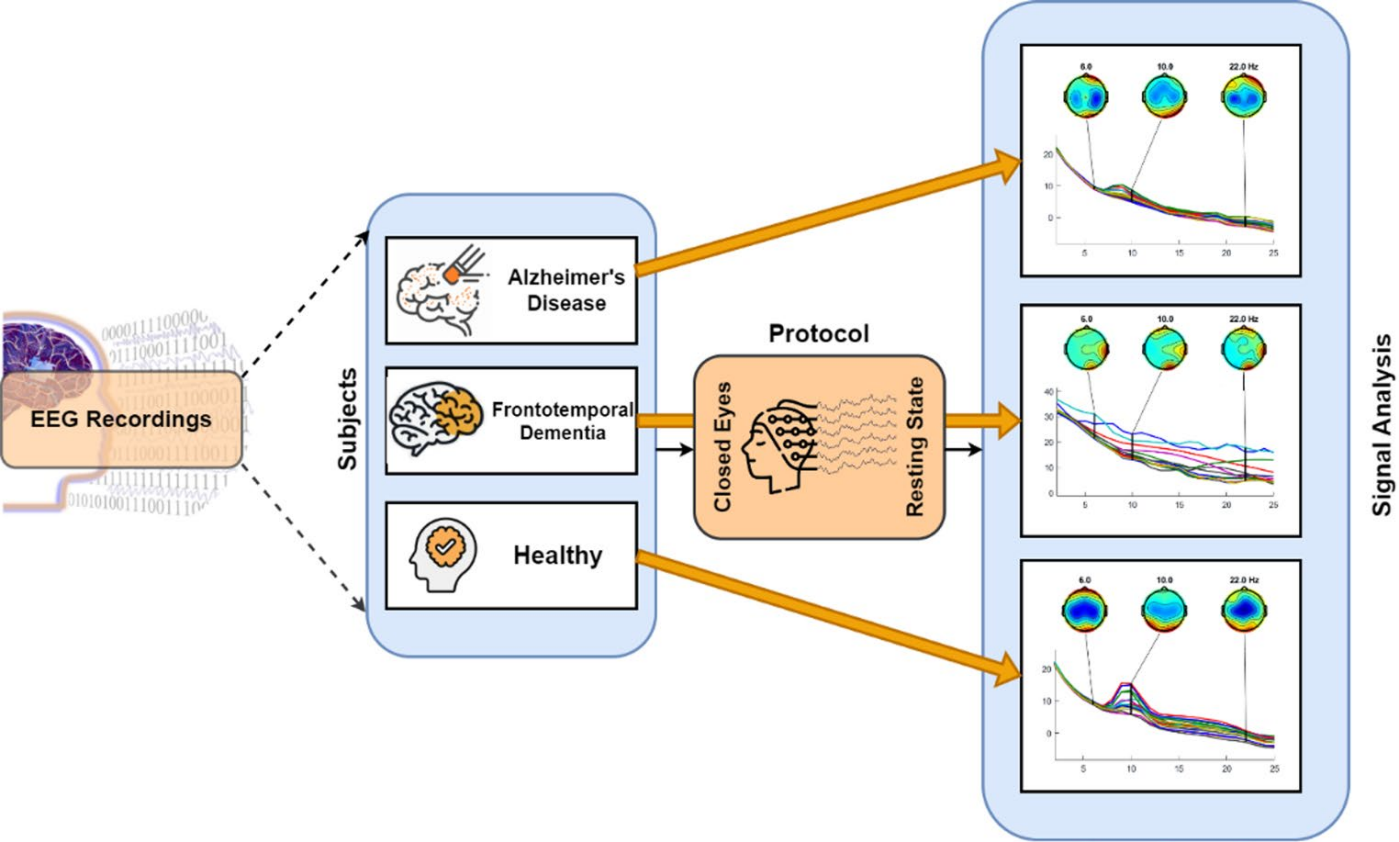
Brief visualization of the AHEPA dataset

Among the few available datasets, the AHEPA dataset (Miltiadous et al. 2024a, b) has become one of the most widely used resources in recent years. Since its publication, it has been cited in dozens of ML studies that attempt to classify AD from EEG. Compared with other datasets, the AHEPA dataset offers several advantages: it provides well-documented clinical annotations, consistent EEG acquisition protocols, and sufficient sample sizes to support subject-level validation. Importantly, its open-access status and deposition in standardized repositories have facilitated broad reuse by the community. For these reasons, the AHEPA dataset (Fig. 2) has effectively become a benchmark in EEG-based dementia research, serving as a reference point against which new methodologies can be compared.

In recent years, there has been an explosion of research activity in the field of EEG-based AD classification. While this surge has undoubtedly stimulated innovation, it has also raised concerns about the overall quality and reliability of the published literature. Recent meta-research in medical AI has shown that although the number of publications has grown exponentially, fewer than 4% of studies perform external validation, and uncertainty quantification is rarely implemented, raising serious concerns about reproducibility (Marconi and Cabitza 2025). Similarly, a cross-disciplinary survey of 22 review papers spanning 17 scientific fields documented at least 294 published studies affected by data leakage, highlighting the systemic nature of this problem in ML-based science(Kapoor and Narayanan 2023). Beyond technical flaws, structural incentives in science often reward quantity over quality, leading to the persistence and spread of poor methods—a phenomenon described as the “natural selection of bad science (*The Natural Selection of Bad Science | Royal Society Open Science*, n.d.).

This pattern is evident in the EEG–Alzheimer’s literature as well, where dozens of new articles are published each year, many of which propose “novel” ML pipelines but report implausibly high performance metrics without thorough validation. As a result, the reported findings vary widely in quality: some studies present carefully designed analyses with robust validation, while others suffer from methodological flaws that inflate accuracy estimates. It is therefore essential to apply critical, yet objective, criteria to evaluate whether a given study should be considered valid. In this context, the choice of validation strategy is one of the most important indicators of methodological rigor. Many studies segment continuous EEG recordings into shorter epochs and apply k-fold cross-validation at the epoch level. When the scientific objective is to assess within-subject reliability or session-level stability, such a design can be appropriate. However, when the goal is case/control classification or subject-level generalization, failing to ensure that all epochs from the same subject remain within a single partition introduces severe data leakage. In that scenario, the classifier is effectively trained and tested on overlapping subject-specific information, leading to artificially inflated performance estimates that do not reflect true between-subject discrimination.

From a methodological standpoint, the primary requirement for case/control EEG classification is the complete absence of subject-level data leakage. This condition is satisfied whenever outer evaluation is performed at the subject level—either through Leave-One-Subject-Out cross-validation (LOSO-CV) or through a train–test split in which each participant is assigned exclusively to either the training or testing partition. In contrast, epoch-level k-fold cross-validation, where epochs are randomly distributed across folds irrespective of subject identity, violates this requirement when used for between-subject inference, because subject-specific structure is shared across partitions.

Among subject-level strategies, important differences remain. A single subject-level train–test split ensures independence but remains sensitive to split-dependent bias: performance may vary substantially depending on which participants are allocated to the test set. Repeated subject-level hold-out or grouped k-fold cross-validation (e.g., k ≥ 5 with subject grouping) reduce this instability by averaging performance across multiple partitions, thereby limiting the risk that results reflect an unusually favorable split. LOSO-CV further maximizes data utilization while preserving strict subject independence, as each subject serves once as the test case.

Consequently, validation strategies that simultaneously (i) enforce subject-level independence, (ii) reduce split-dependent bias through multi-fold or repeated resampling, and (iii) retain multiple observations per subject (e.g., epochs) as distinct samples during learning, provide the most methodologically robust framework for case/control EEG classification. Designs that satisfy only the first condition (e.g., a single subject-level split) improve over epoch-level k-fold but may still yield unstable estimates, whereas designs that violate subject-level independence fundamentally compromise generalizability to new individuals.

Given these issues, the present study undertakes a systematic and stringent evaluation of the existing literature. By categorizing each paper not only on the basis of reported performance, but also on the soundness of its methodology (based on strict pre-established criteria), we aim to provide an objective assessment of the current state of the art. This ensures that performance benchmarks are meaningful and that the community can clearly distinguish between genuine advances and results compromised by flawed validation practices.

In this paper, we present a systematic benchmarking of ML methodologies applied specifically to the AHEPA dataset (Miltiadous et al. 2024b), which has become the most widely used open-access EEG resource for AD research. Our analysis goes beyond a simple comparison of performance metrics. In addition to reporting classification accuracy, F1-scores, and other related measures across different studies, we also examine the methodological rigor of each approach. We highlight the strengths and limitations of their validation practices to provide a more comprehensive evaluation. Furthermore, we provide a comprehensive overview of emerging trends in feature extraction and algorithm selection, mapping how the community has applied traditional classifiers and deep learning models to this dataset. By consolidating this information, the present work provides a clear performance baseline and methodological reference point. This allows new research groups to approach the problem from a computational perspective. In doing so, they can understand not only which performance thresholds need to be surpassed, but also which methodological pitfalls should be avoided.

## Methodology

This section outlines the approach used for the selection of the studies, the screening procedure, the assessment of eligibility, and, lastly, the procedure for analyzing the included research. The study design followed the Preferred Reporting Items for Systematic Reviews and Meta-Analyses (PRISMA) guidelines, guaranteeing transparency and reproducibility in the selection and evaluation of eligible papers, and can be classified as a Scoping Review.

### Description of the AHEPA dataset

We will refer to the dataset used from all the papers in this study as the AHEPA dataset. The dataset consists of resting-state, eyes-closed scalp EEG recordings from 88 participants acquired in a clinical routine setting, including 36 patients with AD, 23 with FTD, and 29 CN age-matched controls. EEG signals were recorded using a clinical Nihon Kohden 2100 system with 19 scalp electrodes placed according to the international 10–20 system, sampled at 500 Hz. Recordings lasted approximately 12–14 min per subject. Cognitive status was assessed using the Mini-Mental State Examination (MMSE), with mean scores of 17.75 (SD = 4.5) for AD, 22.17 (SD = 8.22) for FTD, and 30 for CN participants. The mean age was 66.4 years (SD = 7.9) for AD, 63.6 (SD = 8.2) for FTD, and 67.9 (SD = 5.4) for CN, with both male and female participants represented across groups. The dataset is structured in BIDS format and includes both raw and artifact-cleaned EEG signals (Miltiadous et al. 2024a).

### Data extraction

**Fig. 3:**
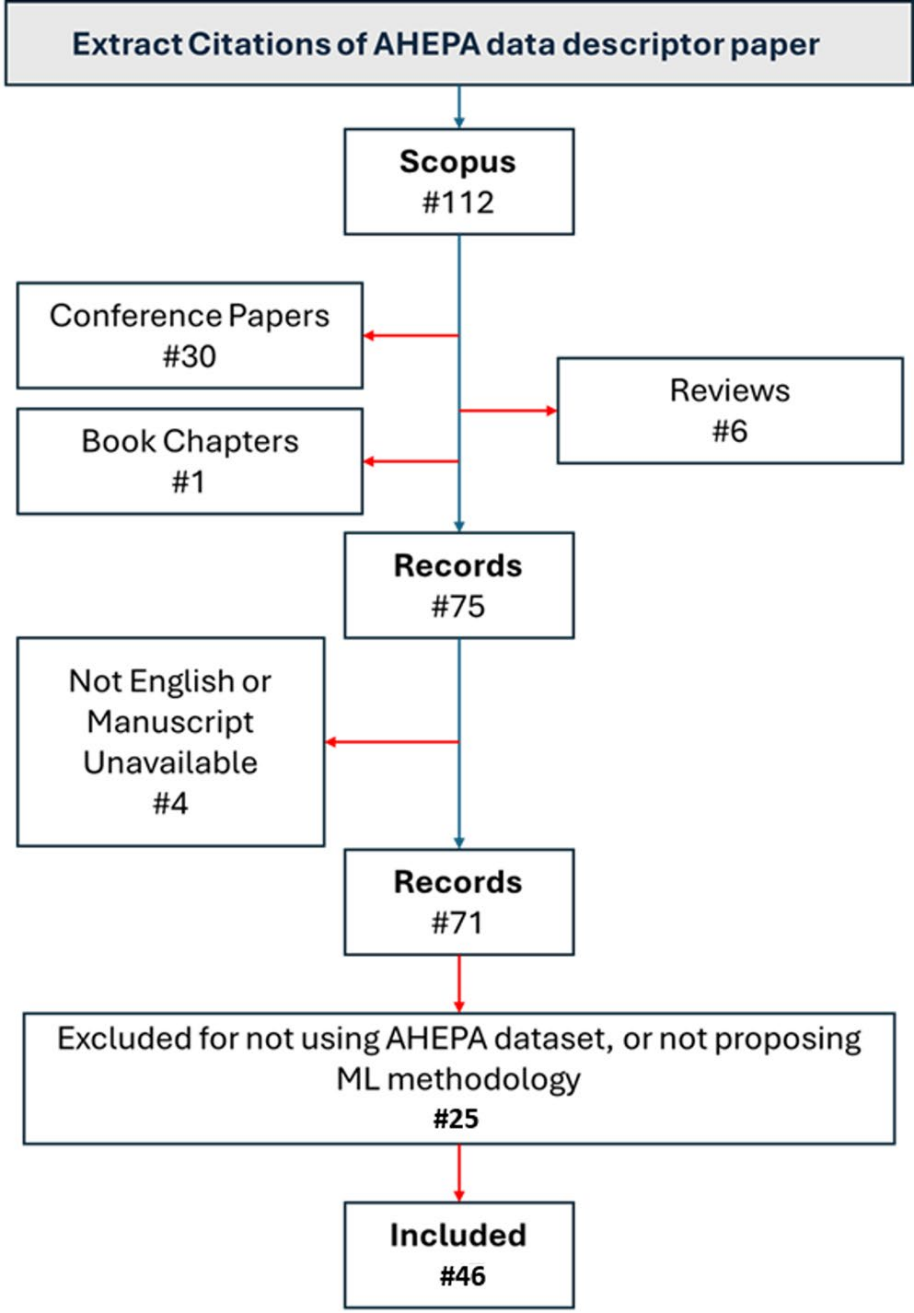
Systematic benchmark review flowchart according to PRISMA statement

The search strategy applied in this paper was designed to capture studies that focus exclusively on ML methodologies for automated classification of AD using EEG data from open-access databases. The protocol for the systematic search of bibliographic records was conducted in accordance with the PRISMA report. This systematic benchmarking of AD classification methods was based on EEG data deriving from the open-access AHEPA dataset. Specifically, the procedure for study selection was as follows: All studies citing the dataset’s data descriptor paper (Miltiadous et al. 2023a, b, c) were retrieved from the Scopus database. Then, all conference papers, reviews, book chapters, and non English manuscripts were removed. Next, all the papers that did not propose an ML methodology for classification of EEG signals or did not use the AHEPA dataset were excluded. The rest of the studies were included in the analysis. The initial search yielded 112 studies. The research and retrieval of the records was on August 26th 2025. All documents were reviewed in full text after duplicates and irrelevant references were eliminated. Studies that used the dataset description merely as a background reference and did not directly apply any ML techniques to the AHEPA EEG data were excluded. The systematic benchmarking was based on the remaining collection of studies. The PRISMA flow diagram depicted in Fig. 3 provides a summary of the research selection procedure.

A total of 112 records were retrieved from the dataset’s Scopus citations. Conference papers, reviews, and book chapters were removed, leaving a total of 75 records. After screening the titles and abstracts to assess the relevance of each study, 4 additional records were excluded for not being written in English or for the manuscript being unavailable. Furthermore, 25 of the 71 papers did not use the AHEPA dataset or they did not propose a ML methodology. Finally, the remaining 46papers were included in this systematic benchmarking.

A structured extraction protocol was applied for each included study, ensuring comparability across studies. For every paper, the following elements were documented in a predefined Excel table:

1. Bibliographic information: paper title, first author.
2. Feature extraction: refers to the kind of input representation that is utilized, such as connection characteristics, Fourier transforms, wavelet transforms, or raw EEG data.
3. ML model: classifiers or deep learning architectures applied.
4. Problem definition: classification tasks performed (e.g., AD vs. CN, AD vs. FTD).
5. Performance metrics: reported results, including accuracy, F1 score, and additional metrics such as sensitivity, specificity, or Area Under Curve (AUC).
6. Validation strategy: details of the evaluation protocol (e.g., cross-validation, train–test split, or subject-level validation such as LOSO-CV).

### Validity assessment

To provide a structured comparison across the included studies, they were divided into three separate validity subgroups. This categorization was important to differentiate studies that employed validated evaluation protocols that were rigorous and free of leakage, compared to manuscripts that described less robust or outright flawed methods. The validity levels imposed aimed to highlight methodological consistency, the validity of reported outcomes and instances in which high performance may be due to design issues versus actual model capability. This distinction allowed us to provide fair benchmark criteria, and unbiased state-of-the-art references.

The validity score for each study was based on explicitly predefined criteria during the data extraction process, using a scale from 1 to 3. In this scale, lower scores indicate higher methodological rigor, with Validity 1 representing the most robust evaluation design and Validity 3 the weakest. Validity 1 refers to studies that used appropriate epoching of EEG data and subject-level validation (e.g. LOSO-CV), which completely mitigated data leakage and produced strong generalizability. In contrast, Validity 2 includes studies that used train–test splits where subjects were not in both groups (thus mitigating leakage) but did not perform repeated resampling, robustness checks, or sufficiently rigorous validation (for example, a single 80/20 split).

Finally, Validity 3 encompasses studies where methodological concerns led to data leakage. Common examples of this category included studies that reported k-fold cross validation at the epoch level rather than the subject level, or cases where feature/channel selection was completed outside of a nested validation.

A substantial effort was devoted to ensuring that validity tier assignment was based exclusively on methodological characteristics explicitly reported in each study. Validity classification relied on predefined structural criteria related to subject-level independence, nesting of model selection procedures, clarity and reproducibility of the validation protocol, segmentation strategy, and statistical treatment of within-subject observations. In cases where methodological descriptions were incomplete or ambiguous, classification was based on the information available in the manuscript text, without inferential assumptions. Any downgrade due to additional methodological concerns not captured by the predefined criteria was explicitly documented.

**Fig. 4:**
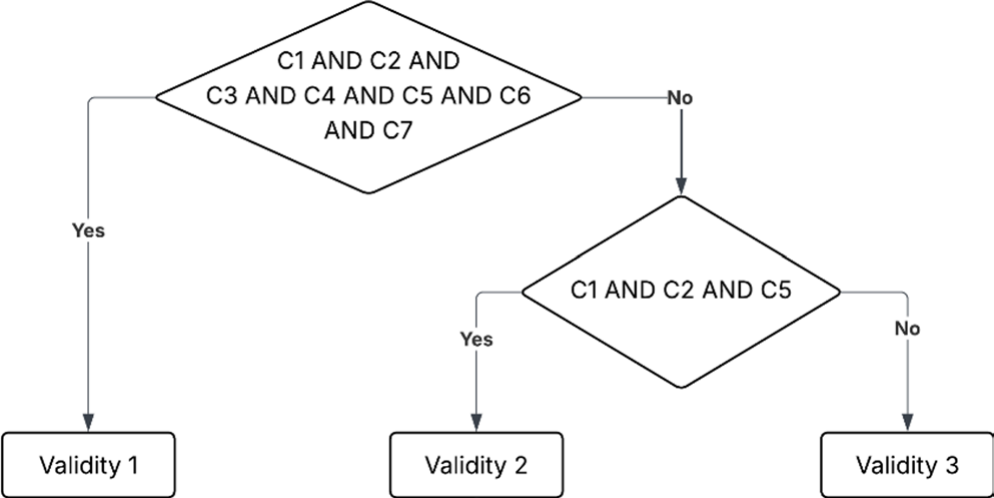
Flowchart of evaluation of the validation category for each paper

To assess the validity group of each paper, 6 criteria were established, named C1-C6, as analyzed in the next paragraph. A study is assigned to Validity 1 (highest methodological rigor) only if all of the criteria are satisfied. A study is assigned to Validity 3 if any of the C1, C2, C5 are not met. A study is assigned to Validity 2 if C1,C2,C5 are met but at least one of the C3, C4, C6, C7 are not met (Fig. 4).

The criteria C1–C7 are:

#### C1

Subject-level independence of outer evaluation.

#### Definition

Whether the outer evaluation protocol (e.g., train–test split or cross-validation) ensures complete subject-level independence between training and testing data, such that no data from the same participant appears in both sets. For example, Yes is: LOSO, or grouped cross-validation/grouped holdout explicitly performed at the subject level. No is: epoch-level or trial-level cross-validation with subjects appearing in both training and testing sets.

#### C2

Fully nested model and feature selection pipeline.

#### Definition

Whether all data-driven steps of the modeling pipeline (including feature selection, channel selection, normalization, dimensionality reduction, and hyperparameter tuning) are performed strictly within the training data of each outer evaluation fold.

#### C3

Signal segmentation and unit-of-analysis definition.

#### Definition

Whether the unit of analysis used for feature extraction and modeling is clearly defined and derived from the EEG signal via explicit temporal segmentation (e.g., epochs or windows), rather than treating the entire recording as a single undifferentiated signal.

#### C4

Transparency of EEG preprocessing.

#### Definition

Whether the EEG preprocessing pipeline is sufficiently described to allow replication, including filtering, artifact handling (e.g., ICA, ASR), referencing, and channel selection.

#### C5

Reproducibility of the validation protocol.

#### Definition

Whether the validation strategy is described with sufficient clarity and detail to allow reconstruction of the evaluation protocol, including the relationship between outer evaluation, inner cross-validation (if any), and subject grouping.

#### C6

Exploitation of within-subject structure.

#### Definition

Whether multiple observations per subject (e.g., epochs/windows) are retained as distinct samples during learning and evaluation, rather than being aggregated (e.g., averaged) into a single subject-level representation prior to modeling.

#### C7

Robustness of outer evaluation scheme.

#### Definition

Whether the outer evaluation design reduces split-dependent bias through multi-fold or repeated subject-level resampling, rather than relying on a single train–test partition. For example, Yes is: The outer evaluation is performed using LOSO, grouped k-fold cross-validation (k ≥ 5), or repeated subject-level hold-out (≥ 10 repetitions), ensuring stability against split-specific bias. No is: A single subject-level train/test split is used without repetition, or the evaluation does not involve multiple folds or repeated resampling.

**Fig. 5:**
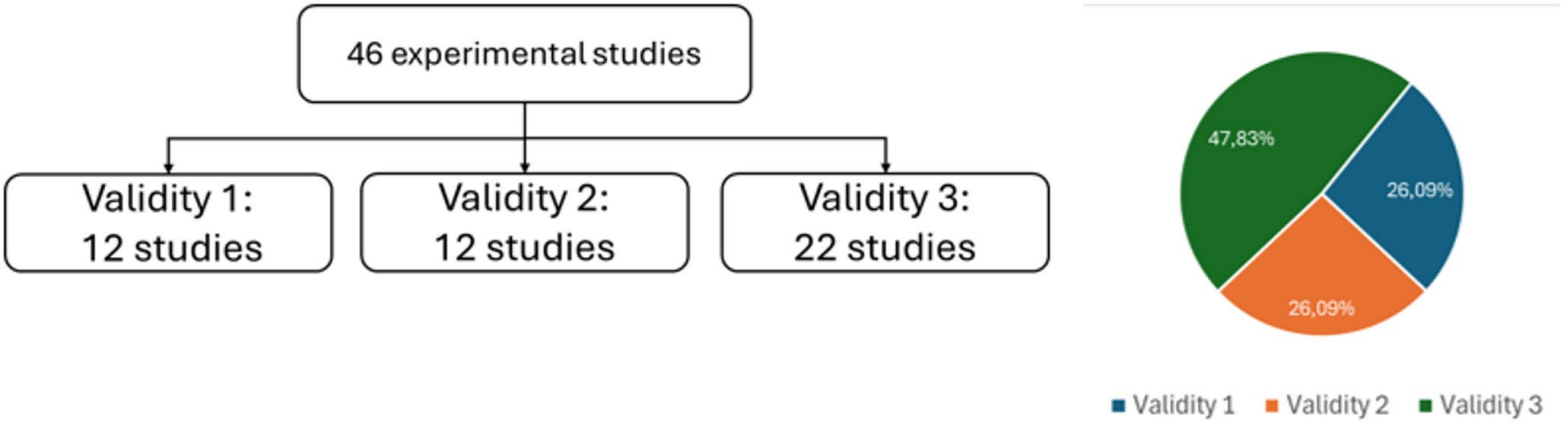
Distribution of the 46 experimental studies across the three validity categories

To perform the validity evaluation regarding the 7 criteria, 2 independent researchers assessed each work. In case of differences in the categorization of a work (Cohen’s κ = 0.79), the reviewers revisited the manuscript and discussed the interpretation of the relevant criteria (C1–C7) until consensus was reached. If necessary, a third senior researcher resolved the disagreement.

### Statistical analysis and benchmarking

All extracted information from the included studies was compiled in a structured spreadsheet, serving as the basis for statistical analyses. The mentioned spreadsheet included the bibliographical information, feature representations, classifiers used, types of validation, reported performance measures, and assigned validity score for each study. The data were then categorized and summarized to provide a quantitative overview of trends and performance benchmarks.

Classification performance was summarized by problem type (e.g., AD versus CN, AD versus FTD, AD and FTD versus CN). For each classification task, accuracy and F1 score mean, were calculated while differentiating by Validity grouping to show methodological design impact. To examine algorithmic trends, classification methods were partitioned into four distinct frameworks: traditional machine learning models (e.g., SVM, Random Forest, Logistic Regression), Convolutional Neural Networks (CNNs), alternative neural architectures (such as RNNs, LSTMs, and Transformers), and ensemble techniques. Approaches utilizing components from multiple categories were classified as hybrid methods.

To quantitatively examine whether methodological rigor systematically affects reported performance, additional statistical analyses were conducted for both the AD–CN and FTD–CN classification tasks. Differences in reported accuracy across validity groups (Validity 1–3) were first assessed using the non-parametric Kruskal–Wallis test, followed by Dunn’s post-hoc pairwise comparisons with Bonferroni correction. To further evaluate the presence of a monotonic relationship between validation rigor and performance, linear regression analysis was performed with reported accuracy as the dependent variable and validity level treated as an ordinal predictor. These analyses were conducted at the study level, using each included publication as a single observational unit.

This systematic procedure allowed not only for the documentation of each study’s reported results but also for the evaluation of their methodological soundness. By combining aggregated statistics with validity-based stratification, a fair and transparent benchmarking of ML approaches applied to the AHEPA EEG dataset was achieved.

## Results

### Overview of included studies

The systematic search and screening process yielded a total of 46 empirical papers that utilized one or more ML methods with the AHEPA EEG dataset to differentiate diagnosis of AD. Collectively, these studies are the largest group of studies that utilize a single open-access EEG dataset as a basis of dementia research to date, highlighting the growing significance of shared resources in dementia research. The overview of studies included in this review is outlined in Table 1. In each row, we include first authors, their primary classifier, their input features, their validation method, and their ultimate validity score. The validity score (1–3) indicates the methodological rigor of each study, with 1 indicating high validity and 3 indicating major methodological inadequacies.

**Table 1:** Summary of the 46 included studies employing machine learning methods on the AHEPA EEG dataset for AD classification

Author	Classifier	Input	Validation Method	Validity
Denghui Zhang (2025) (Zhang and Zhu 2025)	Bi-MCGNN (dual-path GNN with multi-scale convolution + attention fusion)	Raw EEG (5s epochs + 1 s subwindows) + PSD (δ–γ bands) into spatio-temporal & spatial-spectral graphs	LOSO CV	1
Qingjie Xu (2025) (Xu et al. 2025)	MF-MGCN (multi-graph convolutional network)	Differential Entropy (δ, θ, α, β, γ) as node features; adjacency = Pearson correlation (functional) + spatial region connectivity; 10 s segments; 90% overlap; 19 channels	Subject-level 80/20 train–test split; 5-fold CV on training; no subject overlap; not LOSO	3
Tuan Vo (2025) (Vo et al. 2025)	Multi-stage CNN (fusion of spectrogram, scalogram, Hilbert spectrum)	4 s frames with 50% overlap → time–frequency images (STFT, CWT, HHT) processed by CNNs, fusion, subject-level majority voting	LOSO CV	1
Hadis Biglari (2025) (Biglari et al. 2025)	Random Forest, SVM, KNN, Decision Tree	MST graph features from wPLI EEG connectivity (diameter, hierarchy, kappa, LF, EC, BC, degree)	10-fold CV	3
Konstantinos Stefanou (2025) (Stefanou et al. 2025)	Custom CNN (FFT-spectrogram RGB images)	30 s EEG epochs, FFT spectrograms (0.5–45 Hz) into 150 × 150 RGB images	Leave-N-Subjects-Out (LNSO, 5 folds)	1
Zakaria Alouani (2025) (Alouani et al. 2025)	CDB-Net (Dual-Branch CNN, contrastive learning)	Preprocessed EEG (0.5–45 Hz bandpass, ICA, ASR, 1 s epochs)	Subject-level train/val/test split (80/10/10)	2
Sandesh Kalambe (2025) (Kalambe et al. 2025)	SBPFAN (CNN with separable + dilated conv + attention)	EEG (19 channels, 8 s windows with 2 s overlap, 2D image representation)	Subject-independent 10-fold CV	3
Zhikang Chen (2024) (Chen et al. 2024)	ANN on custom SoC (step-function classifier)	FFT spectral features from 14 EEG channels (0.5–50 Hz, ICA, downsampled to 1000 Hz)	Train/Validation/Test split	3
Waqar Khan (2025)(Khan et al. 2025)	TCN + LSTM (hybrid DL)	Modified Relative Band Power (6 bands, PSD), 6 s epochs, ASR + ICA + filters	80/10/10 train/val/test split + 5-fold CV on epochs; SMOTE balancing	3
Akanksha Parihar (2025) (Department of Electronics and Communication, UIT-RGPV, Bhopal, India et al., 2024)	Decision Tree, SVM, KNN, Naive Bayes, Ensemble Bagged Trees, NN	Hjorth parameters (Activity, Mobility, Complexity) + Kurtosis from CWT (Delta–Gamma)	LOSO CV	3
Vinurajkumar S. (2025) (Vinurajkumar et al. 2025)	SVM; Discriminant Analysis; KNN; Naive Bayes; Decision Tree; thresholding	Total Gamma Band Power (30–80 Hz) at frontal electrodes (Fp1, Fp2, F3, F7, Fz, F4, F8); 240 s segment per subject; DFT magnitude-squared; 50 Hz notch filter	Subject-level 80/20 hold-out (single split)	2
Siwei Xie (2025) (Xie et al. 2025)	CNN with attention (Stockwell-CNN)	Stockwell transform time–frequency features (2D matrices)	10-fold CV	3
Ahmad Zandbagleh (2024) (Zandbagleh et al. 2024)	Logistic Regression	Beta-to-Theta entropy ratio (MDE, temporal lobe)	LOSO CV	1
Rundong Jiang (2025) (Jiang et al. 2025)	3D-CNN (Coherence-CNN)	Per-subject averaged coherence matrices (19 × 19 across 5 bands) from 8 s epochs (50% overlap)	LOSO CV	2
Yuan Ma (2024) (Ma et al. 2024)	SVM (sigmoid kernel)	PHI (mutual information) between 13 DMN electrode pairs + age & sex; 60-s first epoch (10 µV discretization); dataset preprocessed with 0.5–45 Hz filter, ASR, ICA	LOSO CV	2
Ehssan Aljanabi (2025) (Aljanabi and Türker 2025)	CNN, ResNet (best: ResNet)	Coherence-based time graph images (171 × T) from sliding windows	Subject-wise k-fold cross-validation	3
Dong-Geun Lee (2025) (Lee & Lee, 2025)	LDA (AD & FTD vs. CN; AD vs. CN); SVM (FTD vs. CN)	Hjorth parameters (Activity, Mobility, Complexity) per channel; 0.5–45 Hz filter, ASR + ICA; 80 s windows with moving length 10–80 s	Stratified 5-fold CV on segmented epochs with per-subject majority voting	2
Xiaowei Zheng (2023) (Zheng et al. 2023)	Random Forest; Decision Tree; SVM	Time-domain (mean, variance, IQR); frequency-domain (relative band powers δ–γ, PSD 0.5–45 Hz); complexity (ApEn, PermEn, SampEn, MSE); synchronization (clustering coeff., char. path length, efficiency, small-worldness)	LOSO CV	2
Miguel Angel Vargas Cruz (2025) (Vargas Cruz 2025)	Random Forest (+ PCA; KMeans label as feature)	Correlation adjacency matrices → DFT features + spectral eigenvalues + KMeans cluster label; disease-specific channel pairs (e.g., AD: O2,Cz; FTD: F7,F3)	Subject-wise validation (details not specified); balanced subsets per task	3
Andreas Miltiadous (2023) (Miltiadous et al. 2023a, b, c)	DICE-Net (dual-input Conv + Transformer Encoder + FFN)	Relative Band Power (δ–γ) + Spectral Coherence Connectivity across 19 channels; 30 s windows (15 s overlap); 0.5–45 Hz band-pass, ASR, ICA; AHEPA ds004504	LOSO CV	1
Talifu Zikereya (2024) (Zikereya et al. 2024)	DAC, KNN, NB, RF, SVM	Relative power in theta, alpha, beta bands	Train-test split (60/40, repeated 1000x, subject level)	1
Shyamal Y. Dharia (2025) (Dharia et al. 2026)	DTCA-Net (Dual-Transformer Cross-Attention)	dPTE connectivity matrices + Differential Entropy (DE), reduced to 6 channels	Subject-stratified 10-fold CV, repeated 30× with subject-level voting	1
Fatma Latifoğlu (2025) (Latifoǧlu et al. 2025)	ANN (best), also SVM	Cross entropy features (CPE, CCE, FCE) from inter-channel connectivity; LASSO for feature selection	5-fold CV	3
Mehran Rostamikia (2024) (Rostamikia et al. 2024)	SVM	Time-domain (mean, variance); Frequency-domain (DWT subband power, total power); Connectivity (cross-correlation, coherence); Complex (KFD, Lyapunov exponent, ApEn)	Train-test split (70/30) + 10-fold CV	3
B. R. Nayana (2025) (Nayana et al. 2025)	RF; 1D CNN; 2D CNN (spectrogram)	PSD band features (mean/median/std across δ–γ); raw 19-ch EEG; Morlet wavelet spectrograms stacked across channels	80/20 train–test split (RF) and Group K-fold (5) on epochs for CNNs	2
Madhav Acharya (2025) (Acharya et al. 2025)	EEGConvNeXt (ConvNeXt-inspired CNN with transformer elements)	CWT spectrogram images from 5 s epochs (19-channel EEG)	Hold-out 80:20 split	3
Shynara Ayanbek (2025) (Ayanbek et al. 2025)	XGBoost, LightGBM, CatBoost, CNN	467 handcrafted EEG features (time, freq, complexity, band ratios, entropy, FD) + raw EEG (19ch × 12 s)	Stratified grouped 5-fold CV + 20% independent test set	1
Yuming Sun (2025) (Sun et al. 2025)	MJANet (ESI + multi-branch joint attention + residual CNN)	ESI-derived multi-angle spatial power maps from CWT (Morlet) in α/β/θ; 10 s windows, 50% overlap; four views concatenated to 224 × 224	LOSO CV	1
Shraddha Jain (2025) (Jain and Srivastava 2025)	PDNFN (weight freezing), compared ConvNet, EEGNet, LMDA-Net	Preprocessed EEG (FLICA, segmentation, spatiotemporal features)	k-fold CV	3
Nisreen Said Amer (2024) (Amer and Belhaouari 2024)	CNNs (GoogleNet, AlexNet, SqueezeNet) on FBFT TF images	FBFT-based EEG TF images (selected channels, 2 s windows, 1.5s overlap)	Train/test split (80/20, subject-level)	2
Souhaila Khalfallah (2025) (Khalfallah et al. 2025)	RF; SVM; KNN; LDA; Logistic Regression; CNN; ChronoNet	PSD (Welch), Bandpower, Shannon entropy, DWT; 10-s epochs; FIR/IIR filters + ICA	80/20 train–test split + 10-fold CV (GroupKFold claimed), per-epoch; not LOSO	3
Amir Hossein Hachamnia (2025) (Hachamnia et al. 2025)	LightGBM (best among ensembles)	Relative Band Power features from 4 s EEG epochs (50% overlap),	Monte Carlo cross-validation with random epoch-level 80/20 splits (repeated), not subject-level	3
Siuly Siuly (2025) (Siuly et al. 2025)	CNN (4-layer) on STFT spectrogram images	STFT spectrograms from 3-s segments; 0.5–45 Hz Butterworth; A1–A2 re-ref; ASR; ICA; resample 256 Hz; images 224 × 224	10-fold cross-validation	3
Raiyan Rahman (2025) (Rahman et al. 2025)	MGFormer (CNN–Transformer hybrid: Multi-Granular Token Encoder + Hybrid Feature Fusion)	Raw multichannel EEG; 5-s epochs (no overlap); 0.5–45 Hz band-pass; A1–A2 re-reference; ASR; ICA; 19 channels	Subject-independent ~ 80/20 subject split;	2
Laura Falaschetti (2025) (Falaschetti et al. 2024)	Lightweight LSTM (2-layer)	EEG spectral representation via DKLT + PCA; 2 s windows with 50% overlap	LOSO CV	1
Yonglin Chen (2023) (Chen et al. 2023)	CNN + Vision Transformer dual-branch with attention	EEG (19 channels, 4 s segments), FFT-based frequency + temporal features, fused CNN/ViT representations	Subject-level 10-fold CV	1
Yujian Liu (2025) (Liu et al. 2025)	MFE-FCGCN (GCN with multi-frequency PSD nodes + Pearson & Mutual Information connectivity)	PSD per band (δ, θ, α, β, γ); multi-frequency graphs; 20 s windows with 75% overlap	Stratified subject-level ~ 80/20 train/test split; 20 repeats; 15-fold CV on training; no segment leakage across sets (not LOSO)	2
Huang Zheng (2024) (Zheng et al. 2024)	SVM	MTRRP features: Recurrence Complexity; Recurrence Rate Gradient; Recurrence Hurst	LOSO CV	1
Prabal Datta Barua (2025) (Barua et al. 2024)	Ensemble kNN with Iterative Majority Voting	EEG (15s segments), N-BodyPat + Attention Pooling features, RFNCA feature selection	10-fold CV	3
Mennato-Allah Talaat Mostafa (2025) (Mostafa et al. 2025)	SVM (RBF kernel)	15 RQA features (Recurrence Rate, Determinism, Entropy, Laminarity, Trapping Time, Transitivity, etc.); compared to Hjorth, Statistical, Relative Power	LOSO CV	3
Xiaoli Yang (2024) (Yang et al. 2024)	SVM; KNN; Random Forest; Logistic Regression	Alpha-band (8–13 Hz) microstate features (duration, coverage, transition probabilities, avg correlation) from 5-s non-overlapping segments; common average reference; microstates via modified k-means	k-fold CV	3
Quoc-Toan Nguyen (2025) (Nguyen 2025)	E-FastKAN (FastKAN + ESN with Decision Tree meta-model)	EEG microstate features (occurrence, coverage, duration, transitions A–E); signals resampled to 200 Hz; 1-min samples (5 per subject)	Cross-dataset evaluation: train 128-sensor BrainLat → test 19-sensor AHEPA; and train 19-sensor AHEPA → test 128-sensor BrainLat; k = 5 CV	3
Pramod H. Kachare (2024) (Kachare et al. 2024)	STEADYNet (lightweight CNN)	Raw EEG windows (4s, 1024 samples, 19 channels), spatiotemporal convolution	60/20/20 train-validation-test split (epoch-level)	2
Zhuyong Wang (2024) (Wang et al. 2024)	SVM (RBF)	Channel-averaged FOOOF aperiodic offsets & exponents + periodic theta power; TAR; PSD (3-min segment per subject); 0.5–45 Hz, ASR, ICA, manual artifact removal	Subject-level 70/30 train–test split; inner 5-fold CV on training; repeated 100 randomized splits; SMOTE	2
Huang Zheng (2025) (Zheng et al. 2025)	SVM	Time–frequency & band-pass functional connectivity features (Pearson r, MI, PLI) from 19-channel EEG	LOSO CV	3
Thawirasm Jungrungrueang (2025) (Jungrungrueang et al. 2025)	CNN (3-stack with dropout, batchnorm, ELU, GAP, FC)	EEG (19 channels, 6 s epochs; ISPC, wPLI, AEC; features: mean, variance, skewness, entropy → connectivity profile maps)	Subject-wise 5-fold cross-validation with SMOTE (no nested CV specified)	3

The table lists the first author, classifier, input features, validation methodology, and the assigned validity score (1–3), reflecting the methodological rigor of each study

The methodological validity of the included studies showed considerable variability. After the validity grouping analysis, 12 studies were categorized as validity 1, 12 studies as validity 2, and 22 studies categorized as validity 3 (see Fig. 5). Validity 1 studies generally followed the best practices, like epoch-based processing plus subject-level cross-validation (e.g., LOSO). In contrast, Validity 3 studies often exhibited methodological issues such as data leakage (e.g., using k-fold cross-validation at the epoch rather than the subject level, or applying train–test splits without justification for subject overlap), inadequate preprocessing, or implausibly high reported performance resulting from poor validation practices.

To provide transparency, Table 2 summarizes the reasoning behind each validity rating. Each entry briefly explains the methodological strengths or weaknesses that justified the assigned score, as well as the criteria not met, according to the validation scheme.

**Table 2:** Overview of the 46 included studies using the AHEPA EEG dataset for Alzheimer’s classification, showing author, classifier, input, validation method, and validity score

Author	Validity	Reasoning	Criteria not met
Denghui Zhang (2025) (Zhang and Zhu 2025)	1	Used LOSO validation, proper preprocessing (ASR + ICA), and 5 s epoching; multiple metrics reported; strong journal and well-structured manuscript. Lack of external cross-dataset testing is a minor limitation.	-
Qingjie Xu (2025) (Xu et al. 2025)	3	Used single subject-level 80/20 split with heavy 90% overlapping augmentation and no LOSO; the very high accuracy on AHEPA (≈ 96%) is likely optimistic relative to stricter subject-level LOSO validation. Fails to satisfy C7; specifically, the outer evaluation protocol relies on a single subject-level hold-out split (80/20 partition) without implementing a multi-fold or repeated resampling strategy to ensure stability against split-dependent bias	C7
Tuan Vo (2025) (Vo et al. 2025)	1	Performed LOSO subject-level validation with epoching and preprocessing (ASR + ICA); reported multiple metrics (Acc, Sens, Spec, F1).	-
Hadis Biglari (2025) (Biglari et al. 2025)	3	Used 10-fold CV on segmented epochs, leading to subject-level leakage;	C1, C2, C5
Konstantinos Stefanou (2025) (Stefanou et al. 2025)	1	Employed LNSO validation, proper preprocessing (ASR + ICA), and 30 s epoching; reported accuracy, precision, recall, and F1.	-
Zakaria Alouani (2025) (Alouani et al. 2025)	2	Explicit subject-independent 80/10/10 train/val/test split with no subject overlap; full metrics reported and robustness tested. No multi-fold or repeated resampling validation scheme, failing to mitigate potential split-dependent bias	C7
Sandesh Kalambe (2025) (Kalambe et al. 2025)	3	Applied subject-independent CV, but the methodology does not clarify how overlapping windows and subject partitioning were handled, creating ambiguity about whether subject-level independence was fully preserved, fails C2, C4	C2, C4
Zhikang Chen (2024) (Chen et al. 2024)	3	The methodology violates C1, C7 by utilizing a randomized 85/15 partition without explicit subject-level grouping, and fails C2 because channel selection (reducing leads from 21 to 14) was performed globally based on experimental performance analysis across the entire dataset prior to the evaluation split.	C1, C2, C7
Waqar Khan (2025)(Khan et al. 2025)	3	Fails C1 because it employs a randomized epoch-level data split (80/10/10) instead of a subject-independent partition, violates C2 violates C2 as the feature normalization process utilized global min and max parameters derived from the entire dataset prior to splitting	C1, C2, C5
Akanksha Parihar (2025) (Department of Electronics and Communication, UIT-RGPV, Bhopal, India et al., 2024)	3	. Employed LOSO validation but, feature selection is not nested inside the training folds, therefore fails C2	C2, C4, C5
Vinurajkumar S. (2025) (Vinurajkumar et al. 2025)	2	Subject-level hold-out, fails C7 by relying on a single static 80/20 split, fails C3 by treating the 240 s recording as an undifferentiated unit, and consequently fails C6 by aggregating all temporal data into a single subject-level feature.	C3, C6, C7
Siwei Xie (2025) (Xie et al. 2025)	3	Used 10-fold CV with epoching, mixing epochs from the same subject across folds, thus fails C1, C7	C1, C7
Ahmad Zandbagleh (2024) (Zandbagleh et al. 2024)	1	Used LOSO validation at subject level with entropy-based EEG features, reported multiple metrics, and avoided leakage, making the study methodologically strong.	-
Rundong Jiang (2025) (Jiang et al. 2025)	2	Epochs were averaged per subject, so effective *N* ≈ 88 while training a 3D‑CNN—strong overfitting risk, fails C6.	C6
Yuan Ma (2024) (Ma et al. 2024)	2	LOSO-CV at subject level with proper preprocessing inherited from AHEPA; But one sample per subject (using only the first 60-epoch), failing C6.	C6
Ehssan Aljanabi (2025) (Aljanabi and Türker 2025)	3	Despite subject-wise CV, internal inconsistencies in segment counts and reporting of the validation protocol were present, failing C4, C5,	C4, C5
Dong-Geun Lee (2025) (Lee & Lee, 2025)	2	Strong preprocessing and interpretable Hjorth features, but validation uses stratified 5-fold on segments with subject-level voting rather than LOSO—risk of leakage; fails C7	C7
Xiaowei Zheng (2023) (Zheng et al. 2023)	2	C4 is not satisfied because the preprocessing pipeline lacks full reproducibility details, particularly regarding the exact referencing scheme after artifact correction and the objective criteria used for ICA component rejection, limiting strict methodological transparency.	C4
Miguel Angel Vargas Cruz (2025) (Vargas Cruz 2025)	3	Fails C2 due to non-nested exhaustive channel selection and retention of best-performing combinations, introducing selection bias. Fails C5 because subject balancing and validation protocol are insufficiently specified, limiting reproducibility and transparency.	C2, C5
Andreas Miltiadous (2023) (Miltiadous et al. 2023a, b, c)	1	Proper LOSO-CV in subject level	-
Talifu Zikereya (2024) (Zikereya et al. 2024)	1	Performed subject-level train–test split with 1000 repetitions, avoiding epoch-level leakage and yielding robust though not perfect classification, making the methodology sound.	-
Shyamal Y. Dharia (2025) (Dharia et al. 2026)	1	Used subject-stratified 10-fold CV repeated 30× with subject-level voting, ensuring no leakage; results are moderate and credible.	-
Fatma Latifoğlu (2025) (Latifoǧlu et al. 2025)	3	Used only 5-fold CV (no LOSO),, fails C1, C7	C1, C7
Mehran Rostamikia (2024)	3	Used subject-level 70/30 split combined with 10-fold CV, which is stronger than simple per-epoch CV but not as rigorous as LOSO; possible risk of data leakage. Fails C2 due to potential selection bias in feature selection (using t-tests outside a nested loop). Furthermore, it fails C3, C6, and C7 as it lacks clear unit-of-analysis definitions and subject-level aggregation, while the absence of a nested validation design results in potentially optimistic performance metrics	C2, C3, C6, C7
B. R. Nayana (2025) (Nayana et al. 2025)	2	Used group 5-fold CV by subject, which avoids epoch leakage, but is weaker than LOSO and inflates performance on small datasets. Fails C6, and C7 as the evaluation is performed per-sample without subject-level aggregation and relies on a validation scheme that is weaker than LOSO.	C6, C7
Madhav Acharya (2025) (Acharya et al. 2025)	3	Used an 80:20 train–test split with 5 s epochs, not LOSO, creating risk of epoch-level leakage. Fails C7. Due to lack of fully nested model and feature selection pipeline fails C2.	C2, C7
Shynara Ayanbek (2025) (Ayanbek et al. 2025)	1	Applied proper subject-level grouped CV with a hold-out test set, avoided data leakage, and explicitly demonstrated how ungrouped CV inflates CNN performance.	-
Yuming Sun (2025) (Sun et al. 2025)	1	Used proper subject-level LOSO with clear epoching and no subject overlap;	-
Shraddha Jain (2025) (Jain and Srivastava 2025)	3	Used epoch-level k-fold CV rather than subject-level validation; Fails C2, C6, and C7 as it lacks a nested validation framework for its complex parameter-freezing mechanism. Furthermore, the performance is reported on a per-epoch basis without subject-level aggregation	C2, C6, C7
Nisreen Said Amer (2024) (Amer and Belhaouari 2024)	2	Although subject-level separation is reported, evaluation relies on a single subject-independent 80/20 split rather than LOSO or repeated subject-level resampling, limiting robustness against split-dependent bias (C7).	C7
Souhaila Khalfallah (2025) (Khalfallah et al. 2025)	3	Used GroupKFold subject-level CV for ML but applied an 80/20 epoch-level split for DL, which mixes data from the same subject across sets;	C1, C7
Amir Hossein Hachamnia (2025) (Hachamnia et al. 2025)	3	Used Monte Carlo CV with random epoch-level 80/20 splits (4 s windows, 50% overlap) instead of subject-level validation, enabling subject leakage and inflating results, fails C1, C2, C7	C1, C2, C3
Siuly Siuly (2025) (Siuly et al. 2025)	3	Fails C1 because tenfold cross-validation is applied after 3-second segmentation without explicit subject-level grouping, allowing segments from the same subject to appear across folds. Fails C7 because evaluation relies on epoch-level tenfold CV rather than subject-level resampling (e.g., LOSO), making results potentially split-dependent and inflated.	C1, C7
Raiyan Rahman (2025) (Rahman et al. 2025)	2	Subject-level separation avoids epoch leakage, but validation is a subject-independent train–test split (not LOSO), fails C7;	C7
Laura Falaschetti (2025) (Falaschetti et al. 2024)	1	Employed subject-level LOSO-CV with proper preprocessing, ensuring robustness and avoiding data leakage.	-
Yonglin Chen (2023) (Chen et al. 2023)	1	Employed subject-level LOSO-CV with proper preprocessing, ensuring robustness and avoiding data leakage.	-
Yujian Liu (2025) (Liu et al. 2025)	2	Fails C7 because the outer evaluation relies on a single 8:2 train–test split; the 15-fold CV is only internal to the training set (train/validation), and the 20 runs keep an identical split, so split-dependent bias is not mitigated via repeated subject-level resampling	C7
Huang Zheng (2024) (Zheng et al. 2024)	1	Employed subject-level LOSO-CV with proper preprocessing, ensuring robustness and avoiding data leakage.	-
Prabal Datta Barua (2025) (Barua et al. 2024)	3	Fails C1, C2, C7 because it used 10-fold cross-validation without subject-level grouping, not fully nested pipeline.	C1, C2, C7
Mennato-Allah Talaat Mostafa (2025) (Mostafa et al. 2025)	3	Fails **C2** because hyperparameter tuning (grid search over cost and gamma) is performed without an explicitly nested cross-validation structure, meaning model selection is not confined strictly within each LOSO training fold, which may introduce optimistic bias.	C2
Xiaoli Yang (2024) (Yang et al. 2024)	3	Fails C1 because the 70/30 train–test split is performed after epoch segmentation rather than at the subject level. Fails C7 because evaluation relies on a single epoch-level partition without subject-level multi-fold resampling.	C1,C7
Quoc-Toan Nguyen (2025) (Nguyen 2025)	3	Fails C1, C7, because k-fold validation with cross-dataset evaluation, data leakage is present.	C1, C7
Pramod H. Kachare (2024) (Kachare et al. 2024)	2	Fails **C7** because it relies on a **single train–validation–test partition** rather than multi-fold or repeated **subject-level** resampling	C7
Zhuyong Wang (2024) (Wang et al. 2024)	2	It fails C7 vecause it used subject-level random 70/30 splits with repeated training, which avoids epoch leakage but is weaker than LOSO;	C7
Huang Zheng (2025) (Zheng et al. 2025)	3	It violates **C2** and **C5** because feature selection (ranking the top 5–50 features based on correlation with group labels) appears to have been performed globally on the entire dataset prior to cross-validation, while it also fails **C6** by averaging multiple 5-second epoch matrices into a single subject-level representation before classification.	C2,C5,C6
Thawirasm Jungrungrueang (2025) (Jungrungrueang et al. 2025)	3	Κey steps (SMOTE, normalization/hyperparameter selection) aren’t explicitly nested within folds (fails C2);	C2

### Diagnostic classification tasks

In addition to the validity classification, the studies included in this review were also organized according to the specific diagnostic problems they addressed. The overwhelming majority of studies focused on the binary classification of patients with AD and CN. This task has become the prevailing benchmark in the field due to its clinical relevance and relative tractability. Overall, binary classification studies accounted for nearly half of all publications identified.

Aside from the AD vs. CN comparison, several studies aimed to explore more complex clinical questions. Multiple studies attempted to differentiate AD from FTD, which illustrates an immediate clinical need to distinguish dementia syndromes that share symptoms. Additionally, a significant number of works focused on the binary classification of FTD vs. CN. A limited but growing number of papers have extended this comparison to multi-class formulations, often using AD vs. FTD vs. CN. Multi-class classifications offer more potential insight into the generalizability of the model’s conclusions, but also represent a more challenging classification problem. An additional common classification approach involved grouping together several dementia conditions (e.g., AD and FTD vs. CN) to assess the ability of EEG-based ML models to generalize across pathological subtypes and to evaluate whether such models can distinguish “dementia” as an umbrella category from healthy brain activity.

Table 3 displays the distribution of problem formulations across the 46 studies included in the review. Overall, the binary classification AD vs. CN remains the principal and most employed classification problem (ensuring studies are comparable). At the same time, moving toward possibly more ambitiously multi-class and differential diagnostic problems truly reflect the heterogeneity and complexity of dementia in the clinic.

**Table 3:** Classification performance across different diagnostic comparisons

Classification problem	Number of studies	Accuracy(%)	Accuracy (Validity 1)
AD vs. CN	40	90.81%	82.11%
AD vs. FTD	12	86.53%	75.18%
AD vs. FTD vs. CN	12	87%	70%
AD & FTD vs. CN	3	92.81%	80.69%

### Performance on AD vs. CN classification

The binary classification of AD patients versus CN controls is the most widely benchmark to which other studies using the AHEPA dataset are compared. This task structure is clinically relevant as it parallels the fundamental clinical challenge of identifying individuals with pathological findings from negative examples representative of healthy aging. It also tends to be more straightforward for researchers, as differential diagnoses are often more complicated and complex.

**Fig. 6:**
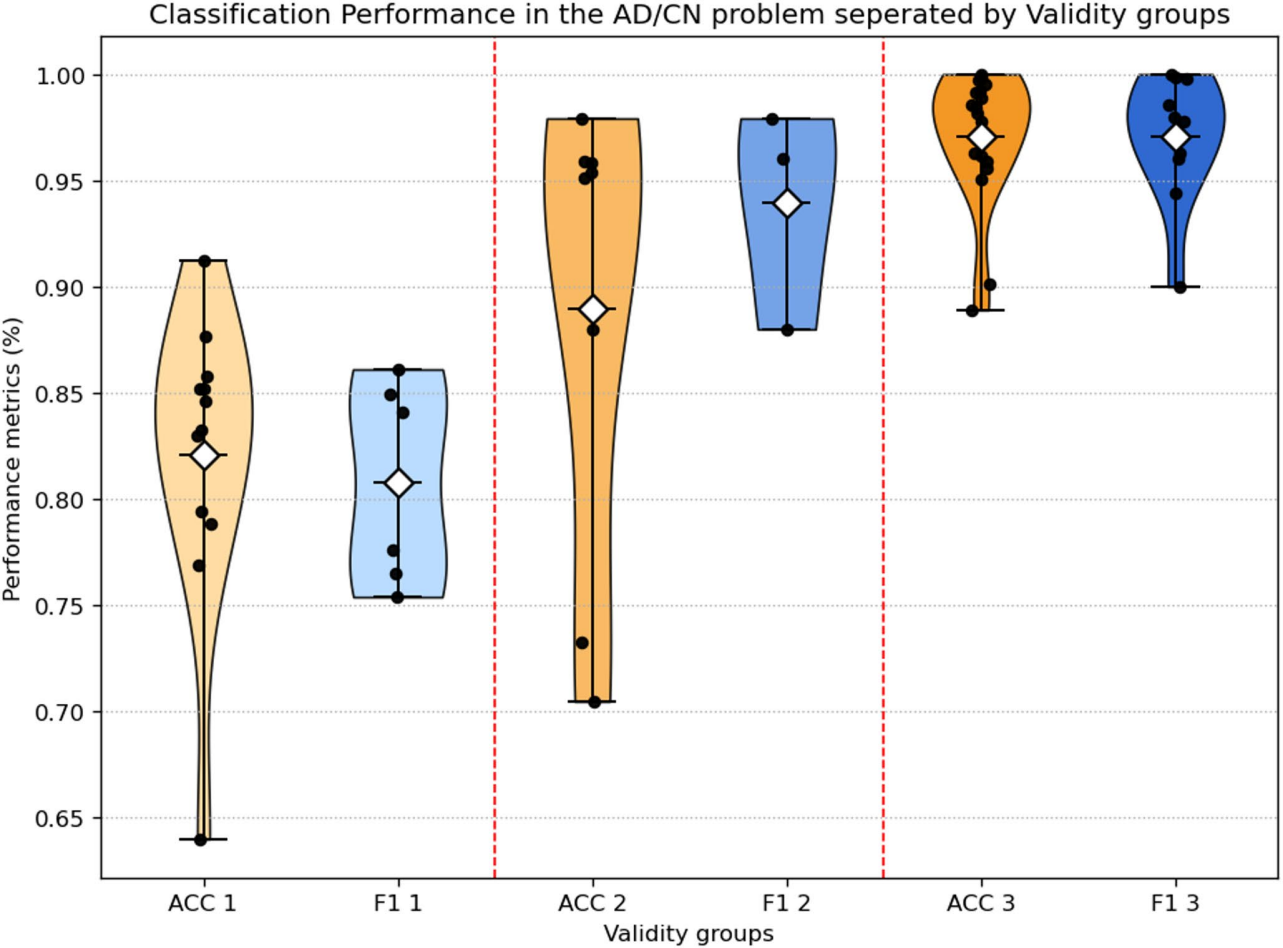
Performance results comparison between the 3 different validity groups for the AD/CN problem

Across the 46 included studies, AD vs. CN was the task studied most often, and reported as part of the study in most the articles. Table 4 summarizes the results of studies on AD vs. CN classification, with the studies presented in an ordered format alongside their respective performance metrics. Furthermore, the differences in performance results between the papers that have been categorized in different validity groups should be noted, highlighting the fact better validity studies often have lower reported performance. This comparison is illustrated in Fig. 6 where the mean Accuracy and F1 score is presented for each different validity group.

**Table 4:** Reported performance of included studies on AD vs. CN classification

Author	Accuracy	F1 Score	Other metrics
Denghui Zhang (2025) (Zhang and Zhu 2025)	91.25%	-	Sensitivity 93.32% Specificity 89.58%
Qingjie Xu (2025) (Xu et al. 2025)	96.15%	98%	Precision 97.67% Recall 98.33% AUC 0.9874
Tuan Vo (2025) (Vo et al. 2025)	84.62%	86.11%	Sensitivity 86.11%, Specificity 82.76%
Hadis Biglari (2025) (Biglari et al. 2025)	100%	100%	-
Konstantinos Stefanou (2025) (Stefanou et al. 2025)	79.45%	77.60%	Precision 77.32% Recall 76.06%
Sandesh Kalambe (2025) (Kalambe et al. 2025)	94.74%	94.64%	Sensitivity 97.98% Specificity 91.53%
Zhikang Chen (2024) (Chen et al. 2024)	98.53%	-	Sensitivity 98.12% Specificity 98.47% PPR 98.42%
Waqar Khan (2025)(Khan et al. 2025)	99.74%	99.76%	Sensitivity 100% Specificity 100%
Akanksha Parihar (2025) (Department of Electronics and Communication, UIT-RGPV, Bhopal, India et al., 2024)	88.90%	-	-
Vinurajkumar S. (2025) (Vinurajkumar et al. 2025)	73.29%	-	-
Ahmad Zandbagleh (2024) (Zandbagleh et al. 2024)	-	-	AUC 0.85 Sensitivity 77.8% Specificity 79.3%
Yuan Ma (2024) (Ma et al. 2024)	76.9%	75.4%	Precision 81.40% Recall 76.90% Specificity 72%
Ehssan Aljanabi (2025) (Aljanabi and Türker 2025)	99.53%	-	AUROC 99.9%
Xiaowei Zheng (2023) (Zheng et al. 2023)	95.86%	-	Sensitivity 96.41% Specificity 97.40%
Miguel Angel Vargas Cruz (2025) (Vargas Cruz 2025)	97%	97%	CN: Precision 100%, Recall 93% AD: Precision 94%, Recall 100%
Andreas Miltiadous (2023) (Miltiadous et al. 2023a, b, c)	83.28%	84.12%	-
Talifu Zikereya (2024) (Zikereya et al. 2024)	83%	-	-
Shyamal Y. Dharia (2025) (Dharia et al. 2026)	85.22%	84.92%	Precision 87.22% Recall 85.28% AUROC 0.83
Fatma Latifoğlu (2025) (Latifoǧlu et al. 2025)	98.46%	98.59%	Sensitivity 97.2% Specificity 100% AUC 99.85
Madhav Acharya (2025) (Acharya et al. 2025)	96.32%	96.32%	Recall 96.44% Precision 96.20%
Shynara Ayanbek (2025) (Ayanbek et al. 2025)	78.87%	76.48%	Precision 87.13% Recall 68.15% ROC-AUC 0.8549
Yuming Sun (2025) (Sun et al. 2025)	85.23%	86.37%	Sensitivity 84.695 Specificity 85.89% Precision 88.12%
Shraddha Jain (2025) (Jain and Srivastava 2025)	95.9%	-	-
Nisreen Said Amer (2024) (Amer and Belhaouari 2024)	95.91%	96%	Precision 96% Recall 96%
Souhaila Khalfallah (2025) (Khalfallah et al. 2025)	99.16%	-	-
Amir Hossein Hachamnia (2025) (Hachamnia et al. 2025)	95.08%	94.39%	Precision 94.57% Sensitivity 94.21% Specificity 95.76% AUC 0.9922
Siuly Siuly (2025) (Siuly et al. 2025)	95.59%	96%	Sensitivity 97.37% Specificity 93.42%
Raiyan Rahman (2025) (Rahman et al. 2025)	70.48%	70.50%	AUROC 0.7851 AUPRC 0.8148
Laura Falaschetti (2025) (Falaschetti et al. 2024)	64%	-	-
Yonglin Chen (2023) (Chen et al. 2023)	85.78%	-	Sensitivity 81.76% Specificity 83.22% AUC 85.88
Yujian Liu (2025) (Liu et al. 2025)	95.09%	94.97%	Precision 93.89% Recall 96.07% AUC 0.9836
Huang Zheng (2024) (Zheng et al. 2024)	87.69%	-	Sensitivity 97.22% Specificity 75.86%
Prabal Datta Barua (2025) (Barua et al. 2024)	99.85%	99.84%	Precision 99.81% Recall 99.88% G-mean 99.87
Mennato-Allah Talaat Mostafa (2025) (Mostafa et al. 2025)	98.20%	-	Sensitivity 99.8% Specificity 96.3%
Xiaoli Yang (2024) (Yang et al. 2024)	99,22%	-	Sensitivity 98.94% Specificity 99.50% AUC 0.9992
Quoc-Toan Nguyen (2025) (Nguyen 2025)	98,88%	-	TPR 0.9848, FPR 0.0073,
Pramod H. Kachare (2024) (Kachare et al. 2024)	88.00%	88.00%	Sensitivity 82.6% Specificity 91.9% AUC 87.2
Zhuyong Wang (2024) (Wang et al. 2024)			
Huang Zheng (2025) (Zheng et al. 2025)	95.38%	-	Sensitivity 94.4%, Specificity 96.6%
Thawirasm Jungrungrueang (2025) (Jungrungrueang et al. 2025)	97.80%	97.80%	-

In total, the overall mean accuracy for all studies conducted on the classification of AD vs. CN was 90.81% (SD = 9.7). The accuracy for the AD vs. CN ranged considerably depending upon the methodological rigor of the validity strategy. For studies rated as Validity 1 (subject-level LOSO or equivalent, *n* = 13), AD vs. CN mean accuracy was more modest and plausible for an AD classification task, averaging 82.11% (SD = 6.93), whereas studies rated as Validity 2 showed (*n* = 8) AD vs. CN mean accuracy was higher than Validity 1, averaging 89% (SD = 11). Conversely, among Validity 3 studies (*n* = 19), when AD vs. CN classification performance was reported, many studies presented “almost perfect” results, with a mean overall accuracy of 97.07% (SD = 3.09)—an implausibly high value given the combinatorial size and complexity of the dataset. These results were very likely to stem from methodological limitations, including epoch-level cross-validation or data leakage, and consequently produced unrealistic and inflated accuracy estimates.

A similar pattern was seen in F1-scores results. The overall AD vs. CN mean F1-score across studies was 90.38% (SD = 9.41), but once again, when stratified by validity category, it revealed differences in average F1-score values. For example, studies rated Validity 1 showed an average F1-score of 81.57% (SD = 4.85), studies rated Validity 2 averaged 89.48% (SD = 7.26), and Validity 3 studies again produced the highest values, with an average F1-score of 97.06% (SD = 2.93), consistent with over-optimistic evaluation protocols.

Overall, the results demonstrate a systematic difference between studies conducted with rigorously validated designs (producing moderate, believable performance estimates) relative to those that were rated methodologically weaker (producing volumes of performance estimates statistically, accruing unrealistically high proposed accuracy’s).

### Performance on FTD vs. CN

**Fig. 7:**
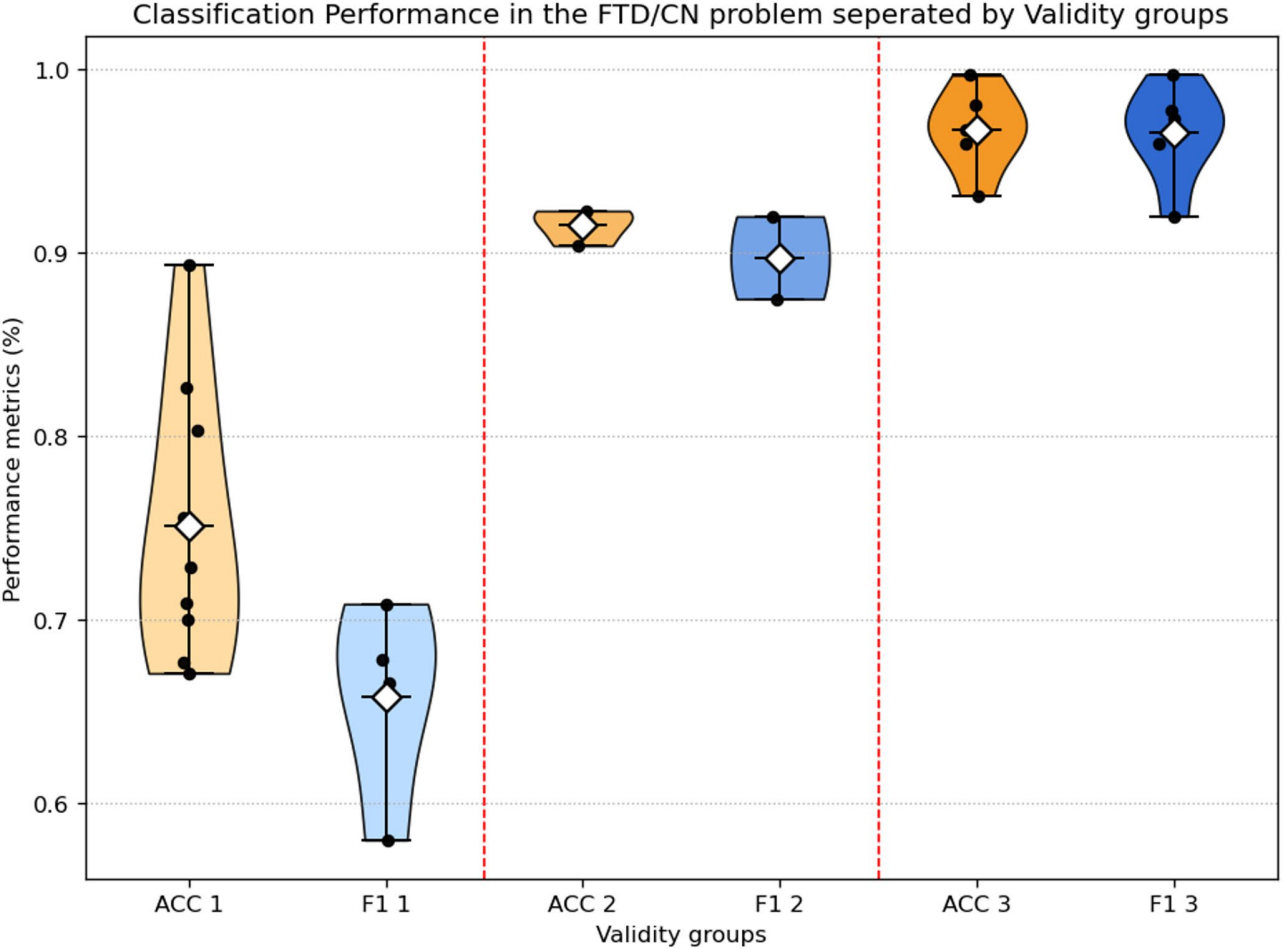
Performance results comparison between the 3 different validity groups for the FTD/CN problem

Among the included studies, the classification of FTD versus CN has been explored less frequently but remains clinically significant. Identifying FTD versus controls helps clarify if EEG-based signatures of dementia include pathological processes that are not limited to AD, and it helps establish a baseline for differential diagnosis tasks that are more complex than AD vs. CN. Table 5 provides an overview of the 25 studies employing FTD vs. CN classification and their corresponding metrics. Again, as discussed in the previous section, the differences between the classification performance for the different validity groups is presented in Fig. 7.

**Table 5:** Reported performance of included studies on FTD vs. CN classification

Author	Accuracy	F1 Score	Other metrics
Denghui Zhang (2025) (Zhang and Zhu 2025)	89.37%	-	Sensitivity 91.82% Specificity 87.81%
Tuan Vo (2025) (Vo et al. 2025)			Specificity < 70%
Konstantinos Stefanou (2025) (Stefanou et al. 2025)	72.85%	67.85%	Precision 71.33 Recall 67.94%
Sandesh Kalambe (2025) (Kalambe et al. 2025)	90.18%	91.12%	Sensitivity 96.61% Specificity 86.22%
Waqar Khan (2025)(Khan et al. 2025)	99.70%	99.75%	Sensitivity 100% Specificity 100%
Ahmad Zandbagleh (2024) (Zandbagleh et al. 2024)	-	-	AUC 0.67 Sensitivity 56.5 Specificity 79.3%
Yuan Ma (2024) (Ma et al. 2024)	90.40%	92%	Precision 91.80% Recall 90.40% Specificity 87.90%
Ehssan Aljanabi (2025) (Aljanabi and Türker 2025)	99.50%	-	AUROC 99.9%
Dong-Geun Lee (2025) (Lee & Lee, 2025)	91.90%		Sensitivity 85.9% Specificity 95.5% AUC 0.950
Miguel Angel Vargas Cruz (2025) (Vargas Cruz 2025)	96%	96%	CN: Precision 100%, Recall 91% FTD: Precision 92%, Recall 100%
Talifu Zikereya (2024) (Zikereya et al. 2024)	70%	-	-
Shyamal Y. Dharia (2025) (Dharia et al. 2026)	67.72%	66.56%	Precision 63.95% Recall 60.06% ROC-AUC 0.82
Fatma Latifoğlu (2025) (Latifoǧlu et al. 2025)	98.10%	97.78%	Sensitivity 95.7% Specificity 100% AUC 0.9971
Madhav Acharya (2025) (Acharya et al. 2025)	98.67%	98.62%	Recall 98.64% Precision 98.60
Shynara Ayanbek (2025) (Ayanbek et al. 2025)	70.92%	58%	Precision 87.13% Recall 68.15% ROC-AUC 0.8549
Yuming Sun (2025) (Sun et al. 2025)	75.57%	70.88%	Sensitivity 70.85% Specificity 78.98% Precision 70.92%
Shraddha Jain (2025) (Jain and Srivastava 2025)	No detailed FTD vs. CN numbers	No detailed FTD vs. CN numbers	No detailed FTD vs. CN numbers
Amir Hossein Hachamnia (2025) (Hachamnia et al. 2025)	99.76%	97.33%	Precision 97.50% Sensitivity 97.16% Specificity 96.15% AUC 99.54
Siuly Siuly (2025) (Siuly et al. 2025)	93.14%	92%	Sensitivity 92.03% Specificity 93.92%
Laura Falaschetti (2025) (Falaschetti et al. 2024)	67.10%	-	-
Yonglin Chen (2023) (Chen et al. 2023)	80.36%	-	Sensitivity 76.34%, Specificity 79.77% AUC 81.77
Huang Zheng (2024) (Zheng et al. 2024)	82.69%	-	Sensitivity 73.91% Specificity 89.66%
Pramod H. Kachare (2024) (Kachare et al. 2024)	92.30%	87.50%	Sensitivity 88.0% Specificity 94.0% AUC 0. 904
Huang Zheng (2025) (Zheng et al. 2025)	86.15%	-	Sensitivity 78.6% Specificity 91.9%
Thawirasm Jungrungrueang (2025) (Jungrungrueang et al. 2025)	96.70%	96.70%	-

The average accuracy across all studies investigating the FTD vs. CN classification problem is 86.53% (SD = 11), and the average F1-score is 86.58% (SD = 3.31). When differentiating the studies by methodological validity, distinct differences in performance became evident. Studies with Validity 1 (rigorous subject-level validation such as LOSO) reported a moderate, but credible mean accuracy of 75.18% (SD = 7.55) and mean F1 of 65.85 (SD = 5.52) which describes the greater difficulty of the differential diagnosis. In contrast, Validity 2 studies—which employed subject-level train–test splits without full cross-subject retesting—reported substantially higher values, with a mean accuracy of 91.53% (SD = 2.71) and a mean F1-score of 92.07% (SD = 4.6). Lastly, studies classified under Validity 3 frequently employed per-epoch k-fold cross-validation or other non-independent data splits and consequently reported near-perfect performance, with a mean accuracy of 94.45% (SD = 5.41) and a mean F1-score of 96.16% (SD = 3.07).

These results demonstrate that, consistent with findings from the AD vs. CN classification task, methodological rigor exerts a strong influence on reported performance. The large disparity between Validity 1 and Validity 3 illustrates how weaker validation strategies can overinflate accuracy and F1-scores, overstating a model’s generalizability to unseen subjects. The more conservative yet realistic outcomes of Validity 1 studies suggest that reliable discrimination of FTD and CN is possible using EEG data, although it still requires substantially more effort than the AD vs. CN classification task.

### Performance on AD vs. FTD classification

The AD vs. FTD classification problem represents one of the most clinically relevant yet challenging comparisons in EEG-based dementia research. Unlike the AD vs. CN task, which contrasts diseased versus healthy subjects, this problem requires distinguishing between two distinct neurodegenerative syndromes that can share overlapping clinical and electrophysiological features. As a result, it serves as a more stringent test of model generalizability and clinical utility.

Table 6 lists the overall results from studies that directly investigated the AD vs. FTD classification task using the AHEPA dataset. The mean accuracy of the studies was 88.97% (SD = 9.92), with an average F1 score of 94.09 (SD = 4.1). Just as observed in the prior benchmarks, the strictness of the validation approach shaped the performance results considerably.

**Table 6:** Reported performance on AD vs. FTD classification using the AHEPA EEG dataset

Author	Accuracy	F1 Score	Other metrics
Ahmad Zandbagleh (2024) (Zandbagleh et al. 2024)	-	-	Sensitivity 75% Specificity 69.6% AUC 0.62
Yuan Ma (2024) (Ma et al. 2024)	96.60%	96.60%	Precision 0.968, Recall 0.966, Specificity 0.947
Ehssan Aljanabi (2025) (Aljanabi and Türker 2025)	99.45%	-	AUROC 0.999
Talifu Zikereya (2024) (Zikereya et al. 2024)	60%	-	-
Fatma Latifoğlu (2025) (Latifoǧlu et al. 2025)	96.61%	95.45%	Sensitivity 91.3% Specificity 100% AUC 0.9939
Mehran Rostamikia (2024) (Rostamikia et al. 2024)	87.80%	-	Sensitivity 85.1% Specificity 90%
Madhav Acharya (2025) [45]	98.21%	98.07%	Precision 97.96% Recall 98.17%
Amir Hossein Hachamnia (2025) (Hachamnia et al. 2025)	94.62%	92.61%	Sensitivity 90.89% Specificity 96.82% Precision 94.40% AUC 0.9867
Huang Zheng (2024) (Zheng et al. 2024)	72.88%	-	Sensitivity 94.44% Specificity 39.13%
Pramod H. Kachare (2024) (Kachare et al. 2024)	89.40%	87.70%	Sensitivity 85.3% Specificity 92.0% AUC 90.1
Zhuyong Wang (2024) (Wang et al. 2024)	-	-	AUC 0.73
Huang Zheng (2025) (Zheng et al. 2025)	84.62%	-	Sensitivity 89.2% Specificity 78.6%

For example, studies using Validity 1 methods (rigorous validation of each subject at the individual subject level (i.e., LOSO)) had a modest mean accuracy of 71.44% (SD = 2.04), and, as with other classifications, this indicates the greater difficulty of making this type of differential diagnosis. Studies using Validity 2 methods had a higher mean accuracy (93, SD = 5.09); while Validity 3 studies reported on accuracy in the near-perfect range (92.82%, SD = 5.81), which are values that likely represent higher but inaccurately inflated values of generalization due to weaker validation schemes or data leakage. However, the significant disparate accuracies reported for the AD versus FTD problem indicate that EEG-based classification can represent disease-specific patterns fairly well when using more advanced representations of the EEG data.

### Performance on AD & FTD vs. CN classification

The AD & FTD vs. CN formulation groups all AD and FTD cases into a single “dementia” category which is then contrasted with CN cases. This is clinically relevant because it assesses whether EEG-based ML can generalize across dementia subtypes to detect neurodegenerative pathology overall, rather than discriminate between specific disease types. Methodologically, this represents an intermediate level of difficulty, more complex than the AD vs. CN task, but not as demanding as a full multi-class classification (AD vs. FTD vs. CN).

Table 7 summarizes the studies on this task. Generally, reported performances were moderate to high, with an average accuracy of 87.1%. The mean accuracy indicates good separation of dementia from controls. However, the results should be interpreted with caution due to the heterogeneity resulting from collapsing AD and FTD into one diagnostic category.

The small number of studies in this category limits generalizability, but some trends were consistent with those observed in the previously reported tasks: studies implementing subject-level validation had reported performance levels with more realistic accuracies (around 75–85%), while studies using simpler train–test splits or cross-validation at the epoch level reported higher performance levels more susceptible to being falsely inflated.

**Table 7:** Reported performance on AD & FTD vs. CN classification using the AHEPA EEG dataset

Author	Accuracy	F1 score	Other metrics
Tuan Vo (2025) (Vo et al. 2025)	-	-	High sensitivity, specificity < 70%
Konstantinos Stefanou (2025) (Stefanou et al. 2025)	80.69%	74.82%	Precision 75.56% Recall 76.15%
Mehran Rostamikia (2024) (Rostamikia et al. 2024)	93.50%	-	Sensitivity 90% Specificity 93%

### Performance on AD vs. FTD vs. CN classification

The AD/CN/FTD task represents a multi-class classification challenge, requiring EEG-based models to simultaneously distinguish among AD, FTD, and CN subjects. This setup provides a more clinically realistic scenario by testing whether models can generalize across different dementia subtypes rather than relying on simple binary contrasts. Multi-class configurations of this kind are generally more difficult, as the EEG markers of AD and FTD often overlap and exhibit substantial intra-subject variability.

**Fig. 8:**
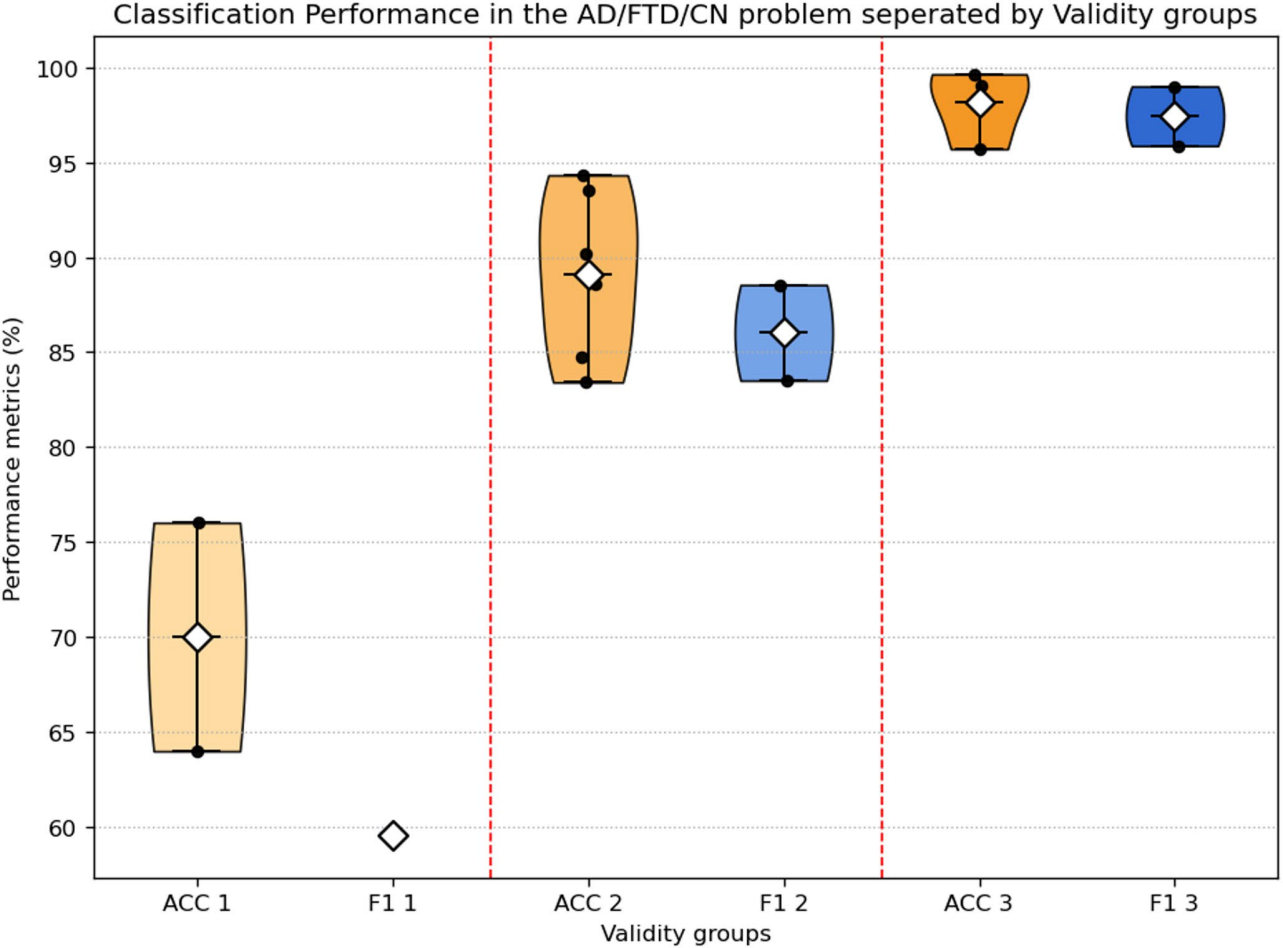
Performance results comparison between the 3 different validity groups for the AD/FTD/CN problem

Table 9 shows the reported classification performance in this three-class context using the AHEPA EEG dataset. For all studies in aggregate, the mean accuracy was 84.29% (SD = 10.72%) and mean F1-score produced was 85.29% (SD = 15.63%). The differences in performance of the validity groups are presented in Fig. 8; Table 8.

**Table 8:** Reported performance on the three-class (AD vs. FTD vs. CN) problem using the AHEPA EEG dataset

Author	Accuracy	F1 score	Other metrics
Yuming Sun (2025) (Sun et al. 2025)	63.97%	59.53%	Sensitivity 60.13% Specificity 80.89% Precision 65.19
Yonglin Chen (2023) (Chen et al. 2023)	76.01%	-	AUC 0.7637
Rundong Jiang (2025) (Jiang et al. 2025)	94.32%	-	Sensitivity 93.28% Specificity 96.92%
Dong-Geun Lee (2025) (Lee & Lee, 2025)	90.20%	-	AUC 0.904
B. R. Nayana (2025) (Nayana et al. 2025)	84.32%	-	-
Thawirasm Jungrungrueang (2025) (Jungrungrueang et al. 2025)	93.50%	-	-
Sandesh Kalambe (2025) (Kalambe et al. 2025)	88.56%	88.54%	-
Zakaria Alouani (2025) (Alouani et al. 2025)	83.41%	83.50%	-
Hadis Biglari (2025) (Biglari et al. 2025)	99.07%	99%	-
Madhav Acharya (2025) [45]	95.70%	95.87%	-
Prabal Datta Barua (2025) (Barua et al. 2024)	99.64%	-	-

When disaggregated by validity, Validity 1 studies (subject-level LOSO) achieved a mean accuracy of 69.99% (SD = 8.51) and F1-score of 59.53%, which reflects the complexity of the task. Validity 2 study results had significantly better performance: 88.25% (SD = 4.36) mean accuracy and 86.02% (SD = 3.56) mean F1-score. Finally, Validity 3 study findings showed highly favorable performance outcomes − 92.64% (SD = 9,84) accuracy and 97.44% (SD = 2.21) F1-score - which are indicative of methodological weaknesses including epoch-level cross-validation, or alertness of subjects.

### Classifier distribution and benchmark performance

The evaluation of classifier use across the 46 studies noted a large variability in methodological decisions. The analysis showed a trend from traditional feature-based classifiers, toward deep learning classifiers with convolutional and graph-based neural networks becoming the most common family of algorithms. Classical ML algorithms such as SVM or Random Forest, continue to be popular, largely due to their ease of use and interpretability, although they are steadily substituted or augmented by architectures that allow direct learning of spatial–temporal patterns found in the EEG data (for instance neural methodologies with incorporated encoder architectures).

To enable a more systematic comparison of methodological performance, the classifiers reported in the included studies were grouped into four overarching families: traditional ML (e.g., SVM, decision trees), CNN-based neural networks, other neural network architectures (e.g., recurrent or graph-based models), and hybrid or ensemble-based approaches. The following subsections discuss the classifier families represented in the included studies, focusing on those appropriate for both EEG data and the AHEPA dataset. A description of these classifier categories is provided in Table 9.

**Table 9:** Description of classifier categories

Classifier category	Description
Traditional ML	SVM, kNN, decision tree, random forest, logistic regression, naïve bayes
CNN-based neural networks	1D/2D/3D CNNs, spectrogram-based deep models
Other neural networks	RNN, LSTM, GNN, MLP, Autoencoder, Transformer variants
Ensemble learning	XGBoost, LightGBM, AdaBoost, Bagging, Stacking
Hybrid architectures	Combination of CNN + Traditional ML, CNN + RNN, Ensemble + CNN, etc. Usage of Transformer architecture components in NN configurations.

### CNN-based neural networks

CNNs (convolutional neural networks) are the most commonly applied class of models in the examined corpus. Their main advantage is their ability to directly learn spatial and spectral representations from EEG-derived images—such as spectrograms, wavelet scalograms, and connectivity matrices—which makes them particularly well suited for modeling time–frequency aspects of neural slowing and topographic power redistribution associated with AD. Table 10 summarizes the studies that employed CNN-based models, along with their reported accuracy for the AD vs. CN classification task and their assigned validity.

Despite the widespread use of CNNs, accuracy among studies differed widely given the validation situation. Studies that employed some form of subject-level validation typically reported accuracies in the 80–85% range, whereas those analyzing epochs using k-fold cross-validation or single train–test splits showed artificially high accuracies (> 95%), suggesting the presence of data leakage. This distinction highlights that validation strategy, rather than model architecture, is one of the most significant factors influencing performance.

**Table 10:** Summary of studies employing CNN-based architectures for EEG-based Alzheimer’s disease detection

First author (Year)	AD vs. CN accuracy	Validity
Tuan Vo (2025) (Vo et al. 2025)	84.62%	1
Konstantinos Stefanou (2025) (Stefanou et al. 2025)	79.45%	1
Yuming Sun (2025) (Sun et al. 2025)	85.23%	1
Zakaria Alouani (2025) (Alouani et al. 2025)	83.41%	2
Nisreen Said Amer (2024) (Amer and Belhaouari 2024)	95.91%	2
Pramod H. Kachare (2024) (Kachare et al. 2024)	88.00%	2
Sandesh Kalambe (2025) (Kalambe et al. 2025)	94.74%	3
Ehssan Aljanabi (2025) (Aljanabi and Türker 2025)	99.53%	3
Thawirasm Jungrungrueang (2025) (Jungrungrueang et al. 2025)	97.80%	3
Siwei Xie (2025) (Xie et al. 2025)	-	3
Madhav Acharya (2025) (Acharya et al. 2025)	96.32%	3
Siuly Siuly (2025) (Siuly et al. 2025)	95.59%	3

### Other neural networks

A small number of studies used neural network architectures outside the traditional CNN frameworks, which we collectively refer to as «Other Neural Networks». Examples include Transformer-based models, Graph Neural Networks (GNNs), lightweight LSTMs, and fusion-based approaches. These architectures are designed to capture long-range temporal dependencies, multimodal relationships, or non-Euclidean spatial structures that may not be fully represented by convolutional architectures.

Transformers and graph-based networks can also model EEG functional connectivity and cross-channel relationships, yielding potentially richer descriptions of the dynamics of the brain networks. Studies using these architectures reported accuracy levels similar to those achieved by CNN-based models, while also offering enhanced interpretability and computational efficiency through attention mechanisms or other explainable components. Table 11 provides a summary of these studies, listing the first author, year, reported accuracy for AD vs. CN classification (if it was available), and the validation strategy or data split used.

**Table 11:** Summary of studies employing neural networks for EEG-based Alzheimer’s disease detection

First author (Year)	AD vs. CN accuracy	Validity
Andreas Miltiadous (2024) (Miltiadous et al. 2023a, b, c)	83.28%	1
Shyamal Y. Dharia (2025)(Dharia et al. 2026)	85.22%	1
Laura Falaschetti (2023) (Falaschetti et al. 2024)	64%	1
Denghui Zhang (2022) (Zhang and Zhu 2025)	91.25%	1
Yonglin Chen (2023) (Chen et al. 2023)	85.78%	1
Raiyan Rahman (2025) (Rahman et al. 2025)	70.48%	2
Yujian Liu (2025) (Liu et al. 2025)	95.09%	2
Qingjie Xu (2025) (Xu et al. 2025)	96.15%	3
Zhikang Chen (2024) (Chen et al. 2024)	98.53%	3
Waqar Khan (2025)(Khan et al. 2025)	99.74%	3
Quoc-Toan Nguyen (2025) (Nguyen 2025)	98.88%	3

### Traditional ML classifiers

Traditional ML algorithms continue to be the method of choice for some studies for using EEG data to detect AD. These studies rely on hand engineered features from EEG data (examples include power spectral ratios, entropy indices, and recurrence quantification) that are fed into classifiers including Support Vector Machines (SVMs), Random Forests (RFs), and Decision Trees. While this approach usually lacks the end-to-end feature learning ability as used in deep neural networks, it is still very popular due to its interpretability, simplicity of computation, and robustness with small datasets.

The accuracy of studies summarized in Table 12 show a diverse range of reported accuracies ranging from 70% to 99% depending on the selected process of feature selection, preprocessing, and validation. Traditional approaches may not model complex spatiotemporal EEG dynamics well, but these studies do provide important interpretive information and seem to function as reasonable baselines as deep learning architectures develop.

**Table 12:** Summary of studies employing traditional ML techniques for EEG-based Alzheimer’s disease detection

First author (Year)	AD vs. CN accuracy	Validity
Talifu Zikereya (2024) (Zikereya et al. 2024)	83.00%	1
Huang Zheng (2024) (Zheng et al. 2024)	87.69%	1
Vinurajkumar S. (2025) (Vinurajkumar et al. 2025)	73.29%	2
Yuan Ma (2024) (Ma et al. 2024)	76.90%	2
Dong-Geun Lee (2025) (Lee & Lee, 2025)	88.60%	2
Xiaowei Zheng (2023) (Zheng et al. 2023)	95.86%	2
Huang Zheng (2025) (Zheng et al. 2025)	95.38%	3
Akanksha Parihar (2025) (Department of Electronics and Communication, UIT-RGPV, Bhopal, India et al., 2024)	88.90%	3
Hadis Biglari (2025) (Biglari et al. 2025)	100.00%	3
Miguel Angel Vargas Cruz (2025) (Vargas Cruz 2025)	97.00%	3
Mennato-Allah Talaat Mostafa (2025) (Mostafa et al. 2025)	98.20%	3
Xiaoli Yang (2024) (Yang et al. 2024)	99.22%	3

### Ensemble models

A limited number of investigations employed ensemble learning strategies in EEG-based diagnosis of AD. Ensemble models aggregate predictions from multiple base classifiers (e.g., neural networks, decision trees, support vector machines) to improve robustness to classification error, reduce the likelihood of overfitting, and improve generalization in heterogeneous EEG datasets. These models exploit the multi-modality of the different architectures to learn complementary EEG representations (both spatially and temporally), and as a result, have very stable predictive performance.

In the three articles identified in the review, ensemble methods achieved very high accuracy (> 95%) in classification of AD vs. CN participants. Specifically, Hachamnia et al. (2025) (Hachamnia et al. 2025) developed an ensemble learning approach in which their framework involved multiple deep and classical models used to classify patients with AD and FTD, with results yielding (95.08%). Barua et al. (2025) (Barua et al. 2024) showed an ensemble based EEG model called N-BodyPat, developed an N-BodyPat based model demonstrated accuracy rates of (99.85%), and appealingly performed along with a strong cross-subject generalization. The summary in Table 13, which has presented the ensemble classification frameworks, has shown that they are among the highest performing measures reported in EEG for dementia classification, although no study was found to be Validity 1 in order to provide an objective reference point.

**Table 13:** Summary of studies employing ensemble models for EEG-based Alzheimer’s disease detection

First author (Year)	AD vs. CN accuracy	Validity
Amir Hossein Hachamnia (2025) (Hachamnia et al. 2025)	95.08%	3
Prabal Datta Barua (2025) (Barua et al. 2024)	99.85%	3
Miguel Angel Vargas Cruz (2025) (Vargas Cruz 2025)	97.00%	3

### Hybrid models

Hybrid models are systems that integrate diverse neural network architectures or other classifiers to leverage the complementary strengths of each approach. These frameworks typically combine convolutional, graph-based, and classical ML components, linking automatic feature extraction with higher-level decision fusion. For instance, Αyanbek (2025) (Ayanbek et al. 2025) proposed a hybrid ensemble and neural network model integrating CNN-based deep learning with boosting, achieving an accuracy of 78.87% for AD detection. Similarly, Jain et al. (2025) (Jain and Srivastava 2025) introduced a hybrid neural network combining CNN and non-CNN components, which attained 95.90% accuracy, while Latifoğlu et al. (2025) [43] developed a non-CNN neural and traditional ML hybrid that reached 98.46%, demonstrating the high discriminative power of multi-paradigm integration.Other studies, such as those by Nayana et al. (2025) (Nayana et al. 2025); Khalfallah et al. (2025) (Khalfallah et al. 2025), explored integrative CNN– ML frameworks, merging deep representations with handcrafted EEG features to improve generalization and mitigate overfitting. Collectively, the hybrid approaches reported in Table 14.

**Table 14:** Summary of studies employing hybrid techniques for EEG-based Alzheimer’s disease detection

First author (Year)	Model/approach	AD vs. CN accuracy	Validity
Shynara Ayanbek (2025) (Ayanbek et al. 2025)	Hybrid Ensemble and Neural Network approaches (CNN-based)	78.87%	1
B. R. Nayana (2025) (Nayana et al. 2025)	Hybrid CNN-based and traditional ML approaches	-	2
Souhaila Khalfallah (2025) (Khalfallah et al. 2025)	Hybrid CNN-based and traditional ML approaches	-	3
Shraddha Jain (2025) (Jain and Srivastava 2025)	Hybrid Neural Networks (CNN + non-CNN)	95.90%	3
Fatma Latifoğlu (2025) (Latifoǧlu et al. 2025)	Hybrid models combining non-CNN Neural Networks and traditional ML classifiers	98.46%	3

**Fig. 9:**
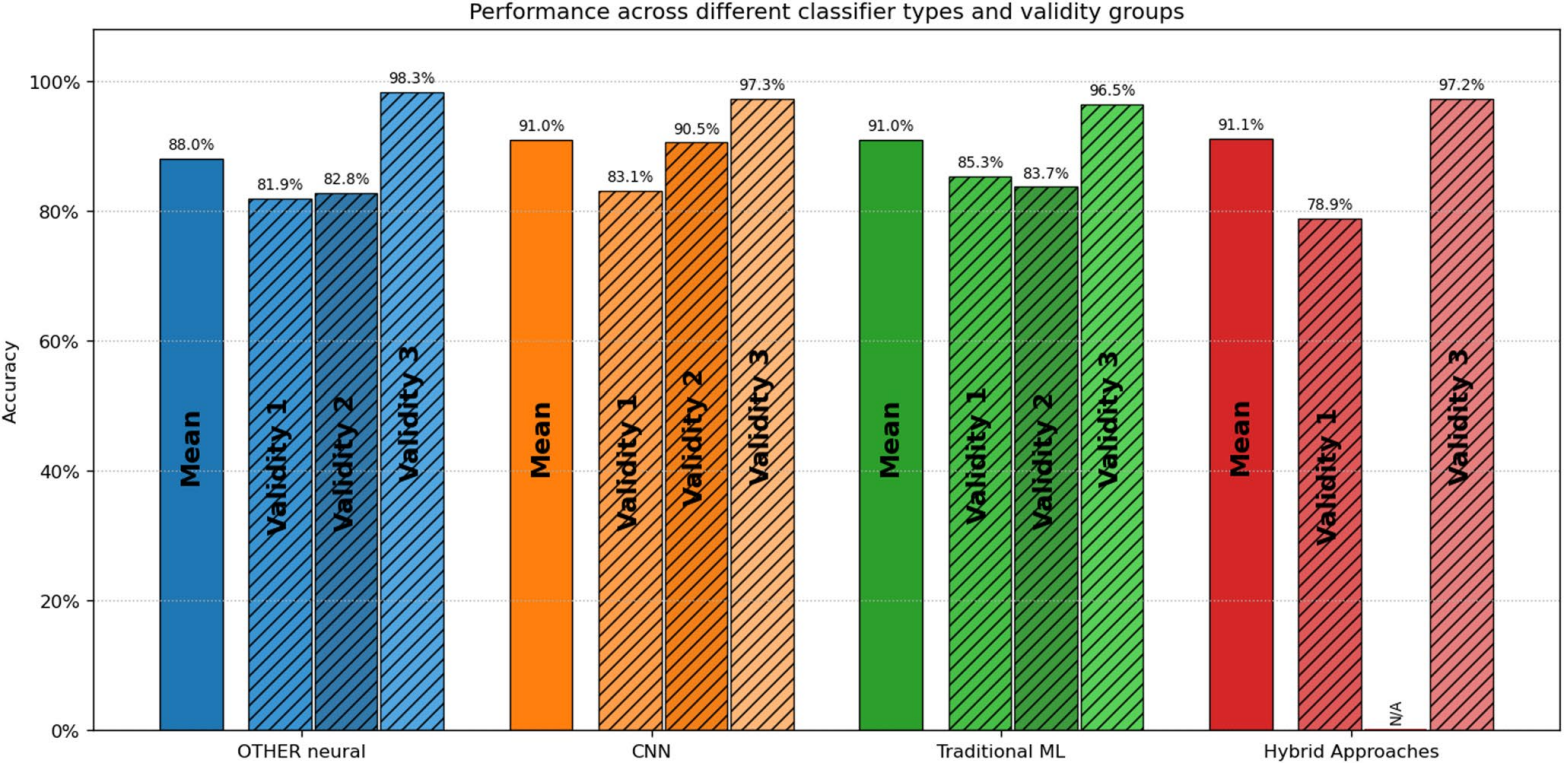
Accuracy of the different ML categories for the AD/CN problem, also separated by their validity group

Figure 9 is presented to better visualize and summarize the performance differences of the different classifier families in the AD/CN problem. The different ML categories are represented in different colors, and the mean Accuracy of them is reported, along with the mean Accuracy for each validity category. We can observe that Hybrid and CNN approaches achieve the best performance overall with 91.1% and 91% Accuracy respectively. For stricter judgment, when taking into consideration only the validity 1 group, traditional ML approaches have reported the best performance so far, with 85.3% average Accuracy.

### Quantitative analysis of the impact of validation rigor on reported performance

For the AD–CN classification task, methodological rigor demonstrated a strong and statistically robust association with reported performance. A Kruskal–Wallis test revealed significant differences in accuracy across validity groups (H = 23.62, *p* < 0.001), with a very large effect size (η² = 0.60), indicating that validation quality accounts for a substantial proportion of performance variance. Post-hoc Dunn comparisons with Bonferroni correction showed that Validity 3 studies reported significantly higher accuracies than both Validity 1 (*p* < 0.001) and Validity 2 (*p* = 0.037) studies, whereas the difference between Validity 1 and Validity 2 was not statistically significant (*p* = 0.367). Pairwise Cliff’s delta values confirmed large to extreme effect sizes, particularly between Validity 1 and Validity 3 (δ = −0.98), consistent with systematic performance inflation in studies employing weaker validation protocols. Complementary linear regression analysis further demonstrated a strong and statistically significant association between validity level and reported accuracy (β = 7.51, *p* < 0.001), with methodological validity explaining 52% of the variance in performance (R² = 0.52). This indicates that each step toward lower validation rigor is associated with an average increase of approximately 7.5% points in reported accuracy.

For the FTD–CN classification task, methodological rigor was again strongly and statistically significantly associated with reported performance. A Kruskal–Wallis test revealed significant differences in accuracy across validity groups (H = 15.23, *p* < 0.001), with a very large effect size (η² = 0.66), indicating that validation quality explains a substantial proportion of performance variance. Post-hoc Dunn comparisons with Bonferroni correction showed that Validity 3 studies reported significantly higher accuracies than Validity 1 studies (*p* < 0.001), whereas differences between Validity 1 and Validity 2 (*p* = 0.157) and between Validity 2 and Validity 3 (*p* = 1.000) were not statistically significant. Pairwise Cliff’s delta values indicated large to extreme effect sizes, particularly between Validity 1 and Validity 3 (δ = −0.96), supporting the presence of marked performance inflation in studies employing weaker validation protocols. Complementary linear regression analysis further confirmed a strong and statistically significant association between validity level and reported accuracy (β = 9.55, *p* < 0.001), with methodological validity explaining 67.6% of the variance in performance (R² = 0.68). These results indicate that each step toward lower validation rigor is associated with an average increase of approximately 9.5% points in reported accuracy.

**Fig. 10:**
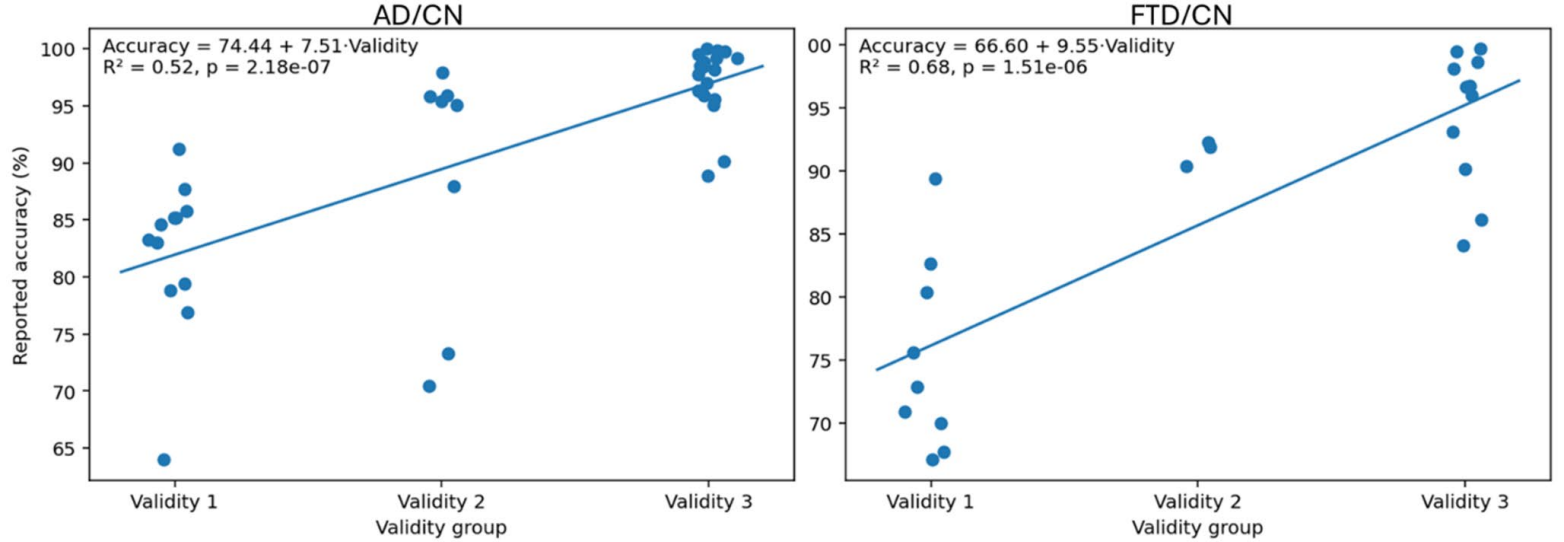
Linear regression analysis illustrating the relationship between validation rigor and reported classification accuracy for the AD–CN (left) and FTD–CN (right) tasks. Each point represents an individual study, and the fitted regression lines demonstrate the positive association between weaker validation protocols and higher reported performance

Figure 10 shows the linear regression between validation rigor and reported classification accuracy across the included studies for the AD/CN (left) and for the FTD/CN (right) problem.

## Discussion

This systematic benchmark provides the most comprehensive quantitative synthesis of EEG-based ML studies employing the open-access AHEPA dataset for dementia classification. Forty-seven empirical papers were reviewed, revealing a highly heterogeneous methodological landscape and substantial variability in reported performance. To objectively compare studies, we implemented a structured validity framework that stratified each paper into three categories according to its evaluation rigor.

- Validity 1 comprised studies that implemented subject-level validation—typically LOSO-CV or equivalent leave-N-subjects-out approaches—together with appropriate epoching, artifact correction (ICA or ASR), and the reporting of multiple complementary metrics. These designs fully prevent inter-subject data leakage and are regarded as the gold standard for reproducible EEG-based benchmarking.
- Validity 2 included studies performing subject-level train/test splits (e.g., 80/20) or limited cross-validation without full LOSO resampling. Although such protocols avoided explicit overlap of epochs between training and testing sets, they generally lacked robustness checks or nested feature selection, resulting in moderately inflated performance.
- Validity 3 encompassed studies using epoch-level k-fold cross-validation, or feature/channel selection performed outside a nested framework, leading to within-subject leakage. These practices systematically overestimate accuracy and account for the majority of the near-perfect (> 95–99%) results reported in the literature.

For future studies, we provide a standardized reporting checklist (Appendix, Table 16), aligned with the C1–C7 framework, to promote reproducibility, clearer reporting, and more reliable evaluation practices in EEG-based machine learning studies. Also, it should be noted that, in the presence of class imbalance, accuracy alone may provide a misleading estimate of performance, and alternative metrics such as F1-score or Matthews Correlation Coefficient (MCC) are generally more informative. Future EEG-based machine learning studies are therefore encouraged to report such metrics alongside accuracy to provide a more complete evaluation.

Importantly, however, even rigorous internal subject-level validation (e.g., LOSO) does not guarantee transportability to independent clinical cohorts, as models may still fail when evaluated on fully external datasets—a phenomenon described as “illusory generalizability” in clinical prediction research (Chekroud et al. 2024).

Across all classification tasks, reported accuracy was inversely proportional to methodological rigor. For the most examined problem—AD vs. CN—the overall mean accuracy across all studies was 90.81% (mean F1 = 81.28%), while the subset of Validity 1 papers achieved a more conservative yet credible 82.5% accuracy and 81.2% F1-score. The best-performing rigorous model was Zhang et al. (Zhang and Zhu 2025), employing a dual-path graph neural network that achieved 91.25% accuracy, while Sun et al. (Sun et al. 2025) reported the highest F1-score among Validity 1 studies (86.4%).

For the FTD vs. CN comparison, the overall mean accuracy was 86.53%, dropping to 76.2% for Validity 1 studies (mean F1 = 66.9%). The highest Validity-1 accuracy in this category was Zhang (Zhang and Zhu 2025) with 89.37% accuracy. The AD vs. FTD differential diagnosis problem showed a mean accuracy of 88.97% across all studies but only 71.44% among Validity 1 papers, again highlighting the methodological inflation characteristic of weaker validation designs. Importantly, this effect was statistically robust, with very large effect sizes (η² up to 0.66), indicating that validation design accounts for a substantial proportion of performance variability.

When dementia subtypes were grouped (i.e., AD + FTD vs. CN), the overall mean accuracy reached 87.1% (mean F1 = 74.82%), yet properly validated models averaged 80.7% accuracy and 74.8% F1, indicating realistic discriminative power for aggregated dementia detection. The best-performing rigorous model in this category was Stefanou et al. (Stefanou et al. 2025), achieving 80.7% accuracy and 74.8% F1. For the more demanding multi-class configuration (AD vs. FTD vs. CN), the overall mean accuracy was 87.0% (mean F1 = 85.29%), but Validity 1 studies averaged only 70.0% accuracy and 59.5% F1; the highest-scoring valid approach was Chen et al. (Chen et al. 2023), reaching 76.0% accuracy, underscoring the increased difficulty of a three class problem.

These findings clearly demonstrate that methodological quality—not model complexity—is the dominant determinant of reported success in EEG-based Alzheimer’s and dementia classification. Only studies that applied more rigorous subject-level validation tended to produce results that appeared more plausible and generalizable, highlighting the value of standardized evaluation protocols and transparent reporting in supporting reliable progress in this growing field. In the following Table 15a summary of the mean performance results for each problem is presented.

**Table 15:** Mean performance metrics for each problem, in total and only regarding the validity 1 group

Problem	Mean ACC	Mean F1	ACC validity 1	F1 validity 1
AD vs. CN	90.81%	90.38%	82.11%	81.57%
FTD vs. CN	86.53%	86.58%	75.18%	65.82%
AD vs. FTD	88.97%	94.09%	71.44%	–
AD + FTD vs. CN	87.10%	74.82%	80.69%	74.82%
AD vs. FTD vs. CN	87.04%	85.28%	69.99%	59.53%

The benchmarking results reveal a clear and systematic relationship between methodological rigor and reported performance. Studies that adopted rigorous subject-level validation (Validity 1) consistently achieved lower but credible accuracies, whereas those relying on per-epoch or loosely defined train–test splits produced inflated results that rarely withstand cross-subject testing. This inverse correlation highlights the central role of evaluation design in shaping the perceived progress of EEG-based dementia research. When performance is assessed under realistic, leakage-free conditions, the classification of AD from EEG stabilizes around the 80–85% accuracy range, establishing a plausible upper bound for current technology.

From an algorithmic standpoint, convolutional neural networks (CNNs) and hybrid deep architectures have achieved the highest nominal accuracies across the literature (mean ≈ 91%), as they can learn rich spatial–spectral features directly from time–frequency images or connectivity matrices. However, when the comparison is restricted to rigorously validated designs, traditional machine-learning approaches—notably support-vector machines and ensemble tree models—perform on par or even slightly better. This convergence suggests that much of the apparent superiority of deep learning arises not from inherent representational advantage, but from data leakage and overfitting to non-independent samples. Consequently, the field’s next step is not necessarily to pursue deeper networks, but to integrate sounder methodological practices and transparent reporting to ensure that claimed gains reflect true generalization.

Nevertheless, graph-based and transformer-based models represent a genuine conceptual advance. Graph neural networks exploit the topological structure of EEG connectivity, modeling long-range dependencies that CNNs cannot easily capture. Even more transformative are transformer architectures, whose self-attention mechanisms can jointly encode spatial, temporal, and spectral relations without explicit feature engineering. Early implementations, such as dual-input convolution-transformer hybrids (Miltiadous et al. 2023a, b, c) and temporal transformers applied to EEG sequences, already appear in the most recent Validity 1 studies of this review, yet their application remains limited by small dataset sizes and the absence of external validation. Looking ahead, the field is expected to evolve rapidly as generative AI reshapes how models are trained and interpreted: pre-training on large unlabeled EEG repositories, fine-tuning for dementia detection, and leveraging foundation or diffusion-based transformer backbones could provide unprecedented generalization and explainability. Although Vision Transformers (ViTs), which process image patches through global self-attention instead of fixed convolutional filters, may offer advantages for EEG spectrogram analysis by capturing distributed spatial–spectral interactions, have not yet appeared systematically within the studies included in this benchmark, their success in other biomedical imaging domains indicates that EEG-spectrogram-based ViTs will likely emerge in forthcoming research. By combining attention-driven feature learning with subject-level validation standards, future transformer frameworks could finally bridge the gap between methodological rigor and state-of-the-art accuracy, marking the next paradigm shift in EEG-based AD classification.

As far as feature extraction is concerned, analysis of the Validity 1 studies indicates that traditional spectral representations constitute a consistent baseline across nearly all works. While a subset of studies incorporates additional EEG biomarkers—such as nonlinear complexity measures or functional connectivity—these extensions do not demonstrate a systematic performance improvement, with all approaches converging to similar accuracy levels (≈ 80–85% for AD vs. CN under subject-level validation). This suggests that spectral features capture the core discriminative information, effectively acting as a de facto reference representation, while more advanced features provide complementary but not consistently superior gains.

A central methodological requirement in EEG-based classification is the use of subject-level cross-validation, typically implemented as LOSO-CV. EEG data are inherently hierarchical, consisting of multiple highly correlated epochs derived from the same recording session. When epochs from a single subject appear in both training and testing partitions, models inadvertently learn subject-specific patterns—such as electrode impedance, noise structure, or individual oscillatory baselines—rather than disease-related neural signatures. This information leakage can artificially inflate performance by up to 15–20% points, yielding results that fail to generalize to unseen individuals. LOSO-CV, applied after epoch segmentation, ensures that all epochs from a given subject are contained within a single fold, thus evaluating the model’s ability to recognize pathology across independent patients rather than memorizing intra-subject variance. In the context of EEG and clinical diagnostics, this makes LOSO-CV not merely a statistical formality but a fundamental criterion of scientific validity and clinical translatability.

Beyond descriptive comparisons, the additional statistical analyses provide quantitative confirmation that validation rigor is a primary determinant of reported performance. Across both AD–CN and FTD–CN tasks, methodological validity explained more than half of the observed variance in accuracy (R² = 0.52 and R² = 0.68, respectively). Moreover, each step toward weaker validation protocols was associated with an average increase of approximately 7–10% points in reported accuracy. These findings indicate that performance inflation is not incidental but structurally linked to validation design. The consistency of the findings across two independent classification problems (AD–CN and FTD–CN) further strengthens the conclusion that validation rigor exerts a systematic and reproducible influence on reported results.

Across the broader EEG–dementia literature, including the studies benchmarked here, a persistent limitation is the lack of generalizability beyond a single dataset configuration. Nearly all published methods—whether built on the AHEPA dataset or alternative institutional collections—are developed and validated on one specific montage, sampling rate, and recording setup, and thus remain tightly coupled to the electrode layout and device characteristics of that source. This single-dataset dependency means that models cannot be readily applied to recordings from different hospitals using distinct EEG systems or alternative electrode configurations, even when they target the same diagnostic classes. As a result, most current pipelines fail to generalize across sites and equipment, substantially limiting their clinical portability. The next critical step for the field is therefore to design cross-configuration adaptive methodologies. For example, robustness could be explicitly evaluated using leave-one-dataset-out or leave-one-montage-out validation schemes, in which models trained on one acquisition setup are tested on recordings obtained with different hardware or electrode configurations. Technically, adaptation may involve spatial interpolation to a common head template, sensor-coordinate–aware graph representations, or domain-adversarial training strategies that reduce device-specific bias during learning. Deep learning models could further integrate learnable alignment layers that automatically adjust to varying electrode topologies during training. To validate such approaches, however, the community urgently needs more open-access, multi-site EEG datasets with standardized metadata and acquisition protocols. Expanding the availability of shared datasets is essential to enable robust cross-hospital testing and to ensure that future classifiers are judged not only by their accuracy but also by their robustness and adaptability across diverse recording configurations—the true prerequisite for clinical translation.

From a clinical standpoint, the realistic accuracies of 80–85% achieved by Validity 1 studies demonstrate that EEG already possesses substantial discriminative power for distinguishing dementia patients from CN individuals, underscoring its potential as a low-cost, non-invasive screening tool. However, differential diagnosis between AD and FTD remains considerably more difficult: when rigorous validation is applied, accuracies typically fall below 70% (Miltiadous et al. 2021), reflecting the overlapping electrophysiological signatures of these syndromes. This limitation points toward the need for multimodal data fusion—combining EEG with MRI, PET, or neuropsychological scores—to improve specificity. Yet, such multimodal approaches are also time- and cost-intensive, precisely the constraints that currently impede large-scale dementia diagnosis and monitoring. The long-term objective is therefore to advance EEG-based methods to the point where they can serve as stand-alone, rapid, and inexpensive screening instruments for early dementia detection, reserving multimodal imaging for confirmatory or differential diagnosis. Achieving this level of clinical utility requires reproducible methodologies and shared open datasets, such as the AHEPA repository, which provide the foundation for cross-site validation and the eventual translation of EEG biomarkers into routine clinical workflows.

### Limitations of this review and future work

This review presents several limitations that should be considered when interpreting its conclusions. First, the proposed validity framework was applied within a domain-restricted context, focusing specifically on EEG-based Alzheimer’s disease versus cognitively normal classification; therefore, its generalizability to other neurological or psychiatric conditions, multimodal pipelines, or longitudinal prediction settings has not yet been established. Second, the review did not perform quantitative re-estimation of reported performance metrics, as no re-analysis of raw datasets or simulation of alternative validation schemes was conducted; consequently, conclusions rely on published results rather than empirically recalculated performance under standardized conditions. Third, although the C1–C7 framework was designed to operationalize methodological validity in EEG machine learning studies, it was not formally compared against established risk-of-bias tools (e.g., structured prediction model appraisal frameworks), and thus its relative coverage and alignment with broader bias taxonomies remain to be systematically examined. Finally, the synthesis was necessarily performance-centric, as most primary studies report classification accuracy (and related discrimination metrics) without sufficient information to evaluate calibration, clinical utility, or downstream decision impact, limiting the scope of interpretability to discrimination-based outcomes.

Future research should therefore proceed along several structured directions. First, there is a clear need for the development of a standardized EEG validity reporting guideline that formalizes requirements for subject-level independence, fully nested model selection pipelines, preprocessing transparency, and reproducible validation protocols. Second, external and cross-dataset validation paradigms must become central to the evaluation process, including cross-cohort and cross-center testing designs that directly assess generalizability beyond single-sample studies. Third, these efforts should be supported by the creation of large-scale, harmonized, multi-center EEG benchmark datasets with predefined evaluation schemes to reduce split-dependent bias and improve comparability across studies. This need is particularly critical given growing evidence that small sample sizes can inflate effect sizes, produce unstable model estimates, and generate non-replicable classification performance, thereby exaggerating apparent group differences (Dede et al. 2025; Ebadi et al. 2025). Larger, systematically curated cohorts are essential to mitigate statistical variance-driven overestimation and to ensure that reported performance reflects robust signal rather than sampling noise. Finally, the field should transition from accuracy-centric competition toward clinically meaningful evaluation, incorporating calibration analysis, decision-analytic metrics, and prospective validation strategies to ensure that reported performance translates into reliable and actionable clinical utility.

## Conclusion

This work establishes the first comprehensive and methodologically validated benchmark of machine-learning approaches for EEG-based dementia classification using the AHEPA dataset. By systematically analyzing 46 published studies through a transparent validity framework, it defines realistic performance boundaries and exposes how methodological rigor—particularly subject-level validation—directly determines the credibility of reported results. Across the AHEPA EEG literature, dementia detection tasks (AD vs. CN and FTD vs. CN) showed high overall reported performance (mean accuracy 90.81% and 86.53%; mean F1-scores 90.58% and 88.58%), yet restricting the analysis to rigorously validated studies (Validity 1) revealed the realistic performance boundaries: 82.11% accuracy and 81.57% F1 for AD vs. CN, and 75.18% accuracy and 65.82% F1 for FTD vs. CN. Within this subset, the strongest AD vs. CN result achieved 91.25% accuracy and 86.37% F1 (different studies), while the best FTD vs. CN performance reached 89.37% accuracy and 70.88% F1 (different studies). The large gap between these credible values and the near-perfect metrics commonly reported in weaker studies underscores the pervasive effect of data leakage, especially when epoch-level cross-validation or non-independent splits are used. By establishing validity-aware performance ceilings, this work provides a methodological standard for future EEG-based dementia classification research. Beyond its immediate benchmarking role, this study serves as a practical foundation for future research: any new methodology applied to the AHEPA dataset should be assessed against the baselines and validity standards introduced here. In this sense, the present benchmark becomes the go-to reference for evaluating methodological soundness and genuine advancement in EEG-based Alzheimer’s and dementia detection, offering a roadmap toward reproducible, generalizable, and clinically meaningful progress in the field.

## Data Availability

No datasets were generated or analysed during the current study.

## Appendix

See Table 16.

**Table 16:** Standardized reporting checklist for EEG-based machine learning studies in dementia

	Checklist table
C1	Have you ensured and explicitly stated that the outer evaluation was performed with complete subject-level separation between training and test data?
C2	Have you ensured and explicitly stated that all data-driven steps (e.g., feature/channel selection, normalization, dimensionality reduction, hyperparameter tuning, class balancing) were performed exclusively within the training data of each outer fold?
C3	Have you ensured and explicitly stated how the EEG was segmented (e.g., epoch/window length and overlap), what each model input sample corresponds to (e.g., one epoch, one window, one subject-level averaged representation, or one full recording), and the number of segments/epochs per subject used for modeling?
C4	Have you ensured and explicitly stated the full preprocessing pipeline, including filtering, artifact handling/removal, referencing, and channel inclusion/exclusion?
C5	Have you ensured and explicitly stated the validation protocol in enough detail to allow reconstruction, including the relationship between outer evaluation, inner validation, and subject grouping?
C6	Have you ensured and explicitly stated whether multiple observations per subject (e.g., epochs/windows) were retained as separate samples or aggregated prior to learning?
C7	Have you ensured and explicitly stated whether the outer evaluation used multi-fold or repeated subject-level resampling (e.g., LOSO, grouped k-fold, repeated grouped hold-out), rather than a single split, and the exact number of folds or repetitions used?
